# Dysregulation of Glucagon Secretion by Hyperglycemia-Induced Sodium-Dependent Reduction of ATP Production

**DOI:** 10.1016/j.cmet.2018.10.003

**Published:** 2019-02-05

**Authors:** Jakob G. Knudsen, Alexander Hamilton, Reshma Ramracheya, Andrei I. Tarasov, Melissa Brereton, Elizabeth Haythorne, Margarita V. Chibalina, Peter Spégel, Hindrik Mulder, Quan Zhang, Frances M. Ashcroft, Julie Adam, Patrik Rorsman

**Affiliations:** 1 Oxford Centre for Diabetes, Endocrinology and Metabolism, Radcliffe Department of Medicine, University of Oxford, Oxford OX3 7LE, UK; 2 Department of Physiology, Anatomy & Genetics, Parks Road, Oxford OX1 3PT, UK; 3 Centre for Analysis and Synthesis, Lund University Diabetes Centre, Department of Chemistry, Naturvetarvägen 14, Lund 221 00, Sweden; 4 Unit of Molecular Metabolism, Lund University Diabetes Centre, Department of Clinical Research in Malmö, Jan Waldenströms Gata 35, Malmö 205 02, Sweden; 5 Nuffield Department of Clinical Medicine, University of Oxford, NDM Research Building, Oxford OX3 7FZ, UK; 6 Metabolic Research, Department of Neuroscience and Physiology, Sahlgrenska Academy, University of Göteborg, Box 433, Göteborg 405 30, Sweden

**Keywords:** glucagon, Fh1, succination, sodium-glucose co-transport, diabetes

## Abstract

Diabetes is a bihormonal disorder resulting from combined insulin and glucagon secretion defects. Mice lacking fumarase (*Fh1*) in their β cells (Fh1βKO mice) develop progressive hyperglycemia and dysregulated glucagon secretion similar to that seen in diabetic patients (too much at high glucose and too little at low glucose). The glucagon secretion defects are corrected by low concentrations of tolbutamide and prevented by the sodium-glucose transport (SGLT) inhibitor phlorizin. These data link hyperglycemia, intracellular Na^+^ accumulation, and acidification to impaired mitochondrial metabolism, reduced ATP production, and dysregulated glucagon secretion. Protein succination, reflecting reduced activity of fumarase, is observed in α cells from hyperglycemic Fh1βKO and β-V59M gain-of-function K_ATP_ channel mice, diabetic Goto-Kakizaki rats, and patients with type 2 diabetes. Succination is also observed in renal tubular cells and cardiomyocytes from hyperglycemic Fh1βKO mice, suggesting that the model can be extended to other SGLT-expressing cells and may explain part of the spectrum of diabetic complications.

## Highlights

• Chronic hyperglycemia inhibits fumarase and glucagon secretion by α cells
• Hyperglycemia causes SGLT-dependent reduction of cytoplasmic pH and ATP production
• SGLT inhibitors normalize cytoplasmic pH, ATP production, and glucagon secretion
• The Na-dependent mechanism may impair ATP production in other SGLT-expressing cells

## Graphical Abstract

**Figure:**
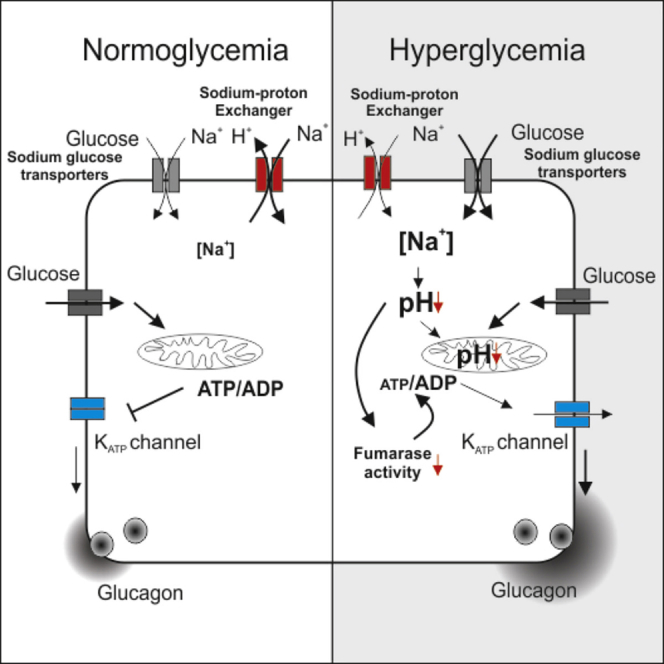

## Teaser

In diabetes, glucagon secretion is dysregulated but the underlying mechanisms are not fully understood. Knudsen et al. report that hyperglycemia impairs glucagon secretion by SGLT-dependent elevation of intracellular Na^+^, leading to acidification, reduced ATP production, and dysregulated K_ATP_ channel activity in α cells. The SGLT mechanism may also impair heart and kidney cell ATP production.

## Introduction

Plasma glucose concentrations are maintained by a tug-of-war between the hypoglycemic effect of insulin and the hyperglycemic effect of glucagon. Under normal conditions, plasma glucose in humans is maintained at ∼5 mM. The benefits of good glycemic control in diabetic patients are well known: it prevents or delays diabetic retinopathy, nephropathy, and neuropathy (Cryer, 2014).

Type 2 diabetes (T2D) results from a combination of insufficient insulin secretion and defective glucagon secretion and culminates in hyperglycemia (Unger and Orci, 2010). T2D affects every cell of the body, which explains the broad range of complications, including accelerated cardiac and renal failure (Forbes and Cooper, 2013). Dysregulated glucagon secretion in T2D manifests as over-secretion under hyperglycemic conditions but insufficient release under hypoglycemic conditions (Dunning et al., 2005, Rorsman et al., 2014). If not alleviated, hypoglycemia results in glucose deficiency in the brain, coma, and ultimately death. In normal situations, hypoglycemia triggers a counter-regulatory response in the α cells (stimulation of glucagon release with resultant increase in hepatic glucose production), but this does not occur in many type 1 diabetes (T1D) and some T2D patients (Cryer, 1998). Patients with T1D experience on average two episodes of symptomatic hypoglycemia every week (Frier, 2009), and it has been estimated that up to 10% of these patients die of iatrogenic hypoglycemia (Skrivarhaug et al., 2006). Therefore, hypoglycemia has been referred to as the limiting factor in diabetes therapy (Cryer, 2002).

Why counter-regulation fails in diabetic patients is not known, but, interestingly, inhibition of mitochondrial ATP production, or pharmacological activation of K_ATP_ channels using diazoxide, recapitulates the dysregulation of glucagon secretion (Zhang et al., 2013). Collectively, these observations suggest that the glucagon secretion defect in diabetic patients is a consequence of disturbed mitochondrial metabolism, but the underlying mechanisms remain obscure.

β cell-specific ablation of the gene encoding the Krebs cycle enzyme fumarase in mice (designated Fh1βKO) results in progressive diabetes. Fh1βKO mice are born normoglycemic and remain so for approximately 8 weeks. Thereafter, they develop hyperglycemia due to loss of glucose-induced insulin secretion (Adam et al., 2017). Here we show that the age-dependent loss of insulin secretion is paralleled by dysregulation of glucagon secretion similar to that in T2D. Similar defects in glucagon secretion develop in other diabetic models: transgenic mice that express a human neonatal diabetes mutation (Kir6.2-V59M) specifically in β cells (Brereton et al., 2014) and Goto-Kakizaki (GK) rats, a model of polygenic diabetes (Granhall et al., 2006).

We have explored the mechanism underlying hyperglycemia-induced dysregulation of glucagon secretion and our data highlight a critical role for Na^+^-glucose co-transport (SGLT)-mediated Na^+^ uptake and intracellular acidification. We propose that this concept may be extended to other cell types and explain part of the spectrum of diabetes-associated complications.

## Results

### Dysregulation of Glucagon Secretion in Fh1βKO Mice

Mice lacking *Fh1* in pancreatic β cells (Fh1βKO) are almost normoglycemic until 10–13 weeks of age (10 weeks in male and 13 weeks in female mice), when they exhibit a rapid and progressive deterioration of glucose homeostasis and insulin secretion (Figures S1A–S1C) (Adam et al., 2017).

**Figure 1:**
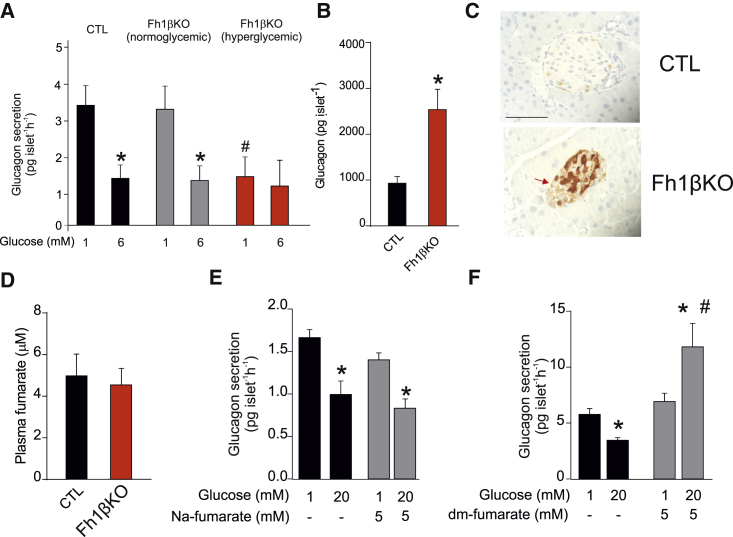
Dysregulation of Glucagon Secretion in Fh1βKO Mice (A) Glucagon secretion in isolated islets from control (CTL; black) and normoglycemic (plasma glucose: <12 mM; gray) and diabetic (plasma glucose: >20 mM; red) Fh1βKO mice at 1 and 6 mM glucose. ^∗^p < 0.05 versus 1 mM glucose; ^#^p < 0.05 versus 1 mM glucose in normoglycemic Fh1βKO islets (n = 8–9 experiments using islets from 12 mice). (B) Islet glucagon content in normoglycemic and hyperglycemic Fh1βKO mice. ^∗^p < 0.05 (n = 12 mice of each group, each measurement based on 12 islets). (C) Immunohistochemistry (IHC) for succination (2SC) in CTL and Fh1βKO islets. Scale bar, 50 μm. (D) Plasma fumarate levels in CTL and severely hyperglycemic (>20 mM) Fh1βKO mice (n = 22 CTL and n = 13 Fh1βKO mice). (E and F) Glucagon secretion in isolated islets from wild-type (NMRI) islets at 1 and 20 mM glucose and supplementing the extracellular medium with 5 mM Na_2_-fumarate (E; n = 4 experiments using islets from three mice), or 5 mM dimethyl (dm)-fumarate (F; n = 12 experiments using islets from four mice). ^∗^p < 0.05 versus 1 mM glucose; ^#^p < 0.05 versus 20 mM glucose. All data presented as mean values ± SEM of indicated number of experiments. See also Figure S1.

We compared glucagon secretion in islets isolated from normo- and hyperglycemic Fh1βKO mice. In islets from normoglycemic Fh1βKO mice, the effects of glucose on glucagon secretion were almost identical to those seen in littermate controls (CTLs) (Figure 1A). In these non-diabetic mice, glucagon secretion was high at 1 mM glucose and inhibited by >60% when glucose was elevated to 6 mM (the concentration associated with maximal inhibition of glucagon release; Walker et al., 2011). However, once hyperglycemia had presented, glucagon secretion at 1 mM glucose was reduced by 60% and elevation of glucose exerted no further inhibitory effect. The reduction of glucagon secretion at 1 mM glucose is remarkable given that glucagon content was increased by 150% in Fh1βKO islets compared with CTL islets (Figure 1B). The increase in content is most likely due to an increase by ∼150% in the proportion of α cells within islets (61% ± 2% cells/islet in hyperglycemic Fh1βKO versus 25% ± 2% cells/islet in CTLs; n = 20 islets from five mice per group; p < 0.001). Thus, glucagon secretion at 1 mM glucose relative to glucagon content is reduced by >80% (from ∼0.33%/hr to 0.06%/hr). In a separate experimental series, we varied glucose between 2 and 20 mM (Figure S1D). Under these conditions, glucagon secretion at 2 mM glucose was reduced by 75% in hyperglycemic Fh1βKO mice compared with CTL mice, and, paradoxically, elevation of glucose stimulated rather than inhibited glucagon secretion, similar to the response of human islets from T2D patients at this high glucose concentration (Walker et al., 2011).

Fumarase catalyzes the hydration of fumarate to malate, and its genetic ablation results in a dramatic increase in intracellular fumarate content (Pollard et al., 2003). Fumarate can react with cysteine residues in proteins to form S-[2-succino]cysteine (2SC), a stable post-translational modification termed succination (Frizzell et al., 2011). We investigated the levels of succination in islets from Fh1βKO by immunohistochemistry with the 2SC antibody. As expected, there was strong 2SC staining in the β cells. However, some succination (albeit lower than in β cells) was also observed in the non-β cells (arrow, Figure 1C; see also Figure 6D). Thus, β cell-specific knockout of *Fh1* also results in elevated fumarate levels in α cells (which are genetically normal).

Plasma fumarate levels were not elevated in hyperglycemic Fh1βKO mice (Figure 1D). Moreover, culturing of wild-type islets with exogenous Na_2_-fumarate for 24 hr did not mimic the effect on glucagon secretion of ablating *Fh1* in β cells (Figure 1E). Thus, the dysregulation of glucagon secretion in hyperglycemic Fh1βKO mice is unlikely to result from “leakage” of fumarate from β cells. However, in islets incubated with membrane-permeable dimethyl-fumarate, glucose not only failed to inhibit but stimulated glucagon secretion (Figure 1F), echoing the changes seen in Fh1βKO mice (Figure S1D). Collectively, these data suggest that ablation of *Fh1* in β cells also increases intracellular fumarate levels in α cells by a systemic effect and this underlies the observed dysregulation of glucagon secretion.

### Ablation of *Fh1* in α Cells Mimics the Effects of Hyperglycemia on Glucagon Secretion

**Figure 2:**
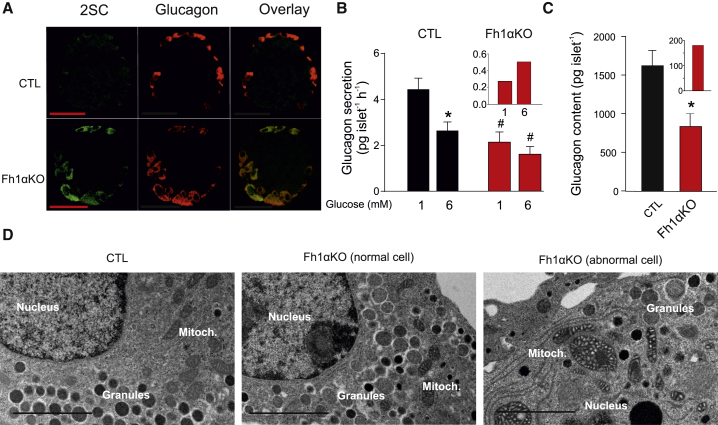
Ablation of Fh1 in α Cells Recapitulates the Effects of Diabetes on Glucagon Secretion (A) IHC for 2SC (green), glucagon (red) and overlay (yellow) islets from CTL (above) and Fh1αKO mice. Note strong 2SC labeling of most glucagon-positive cells. Scale bar, 50 μm. Data are representative of four mice of each genotype. (B) Glucagon secretion in CTL and Fh1αKO islets measured during 1 hr static incubations at 1 and 6 mM glucose. ^∗^p < 0.05 versus 1 mM glucose; ^#^p < 0.05 versus corresponding group in CTL mice (n = 7–8 experiments using islets from three CTL and three Fh1αKO mice). Inset: glucagon secretion at 1 and 6 mM glucose after compensating for α cells that retain *Fh1*. (C) Glucagon content in islets from CTL and Fh1αKO mice. ^∗^p < 0.05 (n = 3 mice for each genotype). Inset: glucagon content after compensating for α cells that retain *Fh1*. (D) Electron micrographs of α cells in a CTL islet (left), a normal α cell in an Fh1αKO islet (center) and an abnormal α cell in an Fh1αKO islet (right; n = 5 cells). Scale bar, 2 μm. All data presented as mean values ± SEM of indicated number of experiments. See also Figure S2.

We generated α cell-specific *Fh1* knockout (Fh1αKO) mice to explore the role of reduced fumarase activity and consequent increased fumarate in the dysregulation of glucagon secretion. Deletion of fumarase, inferred from 2SC labeling, occurred in 58% ± 18% of α cells (n = 4 mice; Figure 2A). Glucagon secretion in Fh1αKO islets at 1 mM glucose was reduced by >40%, and elevating glucose to 6 mM was, unlike the response of CTL islets, not associated with a statistically significant suppression at elevated glucose in Fh1αKO islets (Figure 2B). These changes were associated with a 50% reduction in islet glucagon content (Figure 2C). We corrected (mathematically) glucagon secretion in Fh1αKO mice, to account for the 42% of α cells in which recombination did not occur, by assuming that glucagon secretion by α cells in this model was the same as in CTL islets. This analysis suggests that, in Fh1αKO, the residual glucagon secretion is limited to only ≈15% of that in CTL islets and that increasing glucose from 1 to 6 mM stimulated rather than inhibited glucagon secretion (Figure 2B, inset). Analogously, we estimate that glucagon content in the Fh1αKO mice is reduced by 90%: the 40% of α cells in which recombination did not occur will account for 650 pg/islet (1,629 × 0.4) of the 830 pg/islet in the Fh1αKO. Thus, 180 pg/islet is what can be accounted for by the fraction of α cells that have lost Fh1 (Figure 2C, inset).

In Fh1αKO mice, insulin secretion and content are normal (Figures S2A and S2B) and they exhibit lower plasma glucose levels than CTLs during an intraperitoneal glucose tolerance test (Figure S2C). Insulin tolerance tests did not suggest increased insulin sensitivity that could account for the improved glucose tolerance in Fh1αKO mice (Figure S2D).

Electron microscopy revealed that a subset of α cells in Fh1αKO islets exhibit abnormal ultrastructural morphology, including swollen mitochondria and fewer secretory granules, while others have normal mitochondria comparable with CTL islets (Figure 2D).

### Defective Glucose Regulation of Glucagon Secretion in Hyperglycemic Mice Results from Increased K_ATP_ Channel Activity

**Figure 3:**
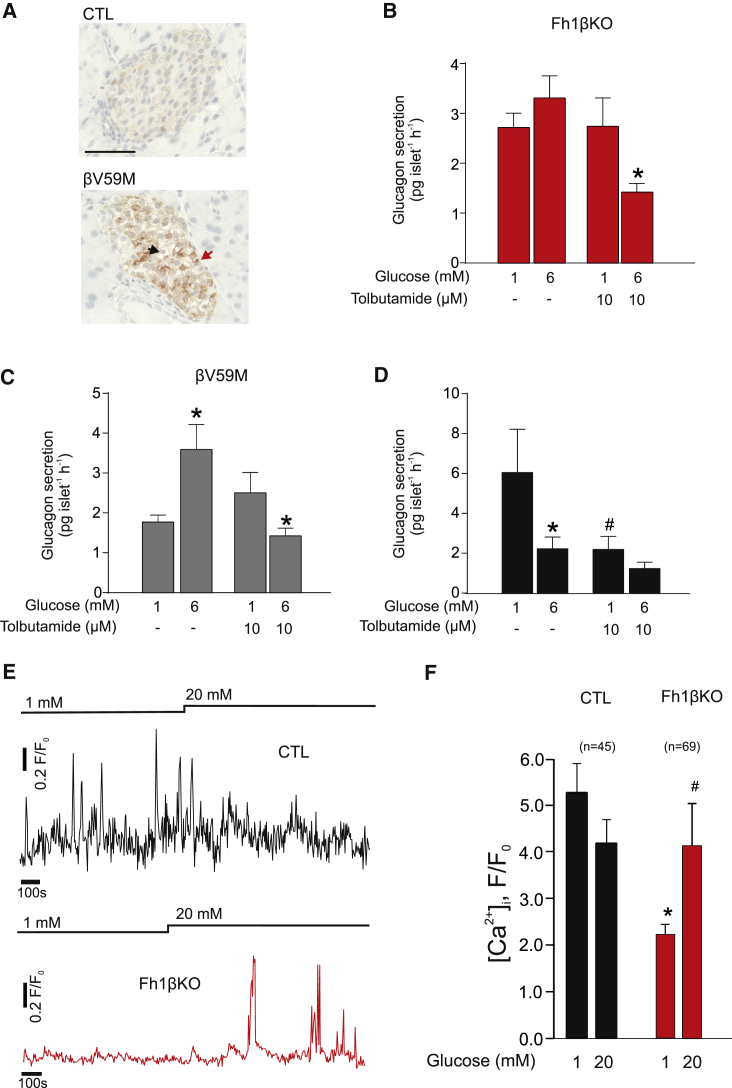
Hyperglycemia-Induced Changes in Glucagon Secretion Are Corrected by Modulation of K_ATP_ Channels (A) IHC for succination (2SC) in CTL and βV59M islets. Scale bar, 50 μm. (B and C) Glucagon secretion in hyperglycemic Fh1βKO (B) and βV59M (C) islets measured during 1 hr static incubations at 1 and 6 mM glucose in the absence or presence of a low concentration (10 μM) of the K_ATP_ channel blocker tolbutamide. ^∗^p < 0.05 versus 1 mM glucose (n = 7–8 experiments using islets from at least three Fh1βKO and three βV59M mice). (D) As in (B) and (C), but experiments obtained from CTL littermates of (n = 6 experiments/6 mice) Fh1βKO and βV59M mice (n = 6–12 experiments/6 mice). ^∗^p < 0.05 versus 1 mM glucose, ^#^p < 0.05 versus 1 mM glucose without tolbutamide. (E) [Ca^2+^]_i_ measurements in α cells, using the calcium dye fluo4, within CTL (black) or hyperglycemic Fh1βKO (red) islets at 1 and 20 mM glucose as indicated. In both genotypes α cells were identified by the [Ca^2+^]_i_ response to adrenaline. (F) Histogram summarizing effects of increasing glucose on [Ca^2+^]_i,_ measured using fluo4, in α cells from normoglycemic CTL (black) and hyperglycemic Fh1βKO (red) islets as indicated. Data are presented as area under the curve. ^∗^p < 0.05 versus 1 mM glucose in CTL mice. ^#^p < 0.05 versus 1 mM glucose in Fh1βKO mice. Data are from indicated number of cells (n) in three islets from one CTL mouse and five islets from two Fh1βKO mice, respectively. All data presented as mean values ± SEM of indicated number of experiments. See also Figure S7.

We hypothesized that the dysregulation of glucagon secretion in the Fh1βKO mice is a consequence of hyperglycemia. If this is the case, glucagon secretion defects similar to those observed in Fh1βKO mice should also develop in other models of hyperglycemia. We tested this using an inducible mouse model of neonatal diabetes with β cell-specific expression of an activating K_ATP_ channel mutation (Kir6.2-V59M) (βV59M) (Brereton et al., 2014). Nutrient-stimulated insulin secretion was switched off in βV59M mice at 12–14 weeks of age. After 14 days of hyperglycemia, α cells in βV59M islets showed similar levels of protein succination as α cells in islets from hyperglycemic FH1βKO mice (compare Figures 1C and 3A; see also Figure 6).

Similar to Fh1βKO mice, glucagon secretion in βV59M islets showed abnormal glucose regulation: glucagon secretion at 1 mM glucose was low and stimulated rather than inhibited by 6 mM glucose. In both Fh1βKO and βV59M islets, normal glucose regulation of glucagon secretion was restored in the presence of 10 μM tolbutamide, a blocker of ATP-sensitive K^+^ (K_ATP_) channels (Figures 3B and 3C). This concentration will block approximately 50% of K_ATP_ channel activity (Trube et al., 1986). In islets from normoglycemic CTL mice, application of 10 μM tolbutamide lowers glucagon secretion at 1 mM glucose by ∼40% and elevation of glucose no longer produces a statistically significant reduction of glucagon secretion (Figure 3D).

These data suggest that the glucagon secretion defect in hyperglycemic βV59M and Fh1βKO mice results from a small increase in K_ATP_ channel activity; if the increase was dramatic, then the low concentration of tolbutamide used would not be able to modulate glucagon secretion. The effects of tolbutamide are not mediated by stimulation of insulin secretion and there were no statistically significant effects of tolbutamide on insulin secretion in Fh1βKO islets (Figure S1E). If K_ATP_ channel activity is increased in α cells from hyperglycemic Fh1βKO mice, then electrical activity should be reduced, similar to what is produced by pharmacological (using diazoxide) or genetic activation (by expression of the V59M gain-of-function mutation) (Zhang et al., 2013). Direct electrophysiological measurements are difficult in diabetic Fh1βKO mice and recordings of the ATP/ADP ratio require prior infection and culture of the islets. Therefore, we used α cell intracellular calcium ([Ca^2+^]_i_) measurements as a proxy for electrical activity (and the cytoplasmic ATP/ADP ratio). We identified α cells in islets from CTL and Fh1βKO mice by the spontaneous [Ca^2+^]_i_ oscillations at 1 mM glucose and responsiveness to adrenaline (Hamilton et al., 2018). In the CTL α cells, [Ca^2+^]_i_ was only marginally reduced by increasing glucose from 1 to 20 mM (Figure 3E), but this effect did not reach statistical significance (Figure 3F). When the same type of measurements were repeated in hyperglycemic Fh1βKO mice, [Ca^2+^]_i_ was reduced by 60% and increased by 150% in response to an elevation of glucose to 20 mM (Figures 3E and 3F). Collectively, these observations suggest that the cytoplasmic ATP/ADP ratio is reduced in α cells from hyperglycemic Fh1βκO mice, leading to the increase in K_ATP_ channel activity that accounts for the inverted response to glucose.

### Hyperglycemia Results in Intracellular Acidification of α Cells

The finding that K_ATP_ channel closure could correct the dysregulated glucagon secretion in hyperglycemic βV59M and Fh1βKO mice suggests that ATP production in the α cell was compromised. Because succination was observed in α cells in both mouse models, we speculated that the reduced ATP production could be linked to increased fumarate caused by a reduction in fumarase expression. However, fumarase was detectable by immunocytochemistry in α cells of Fh1βKO mice (Figure S2E). Thus, reduced expression of *Fh1* is unlikely to explain the glucagon secretion defect and the protein succination in α cells from Fh1βKO mice.

The mitochondrial matrix is alkaline, and Krebs cycle enzymes typically have their maximum catalytic activity at high pH (Bernstein and Everse, 1978, Lai and Cooper, 1986, Willson and Tipton, 1980). We confirmed that this was also the case for fumarase: there was a strong reduction of fumarase activity when pH lowered from 7.6 to 7 and 6.6 in a cell-free system (Figure S2F). At the lowest pH, fumarase activity was only 5% of that seen at pH 7.6. Therefore, we conclude that the reduced fumarase activity in α cells may be a consequence of intracellular acidification rather than lowered gene expression.

**Figure 4:**
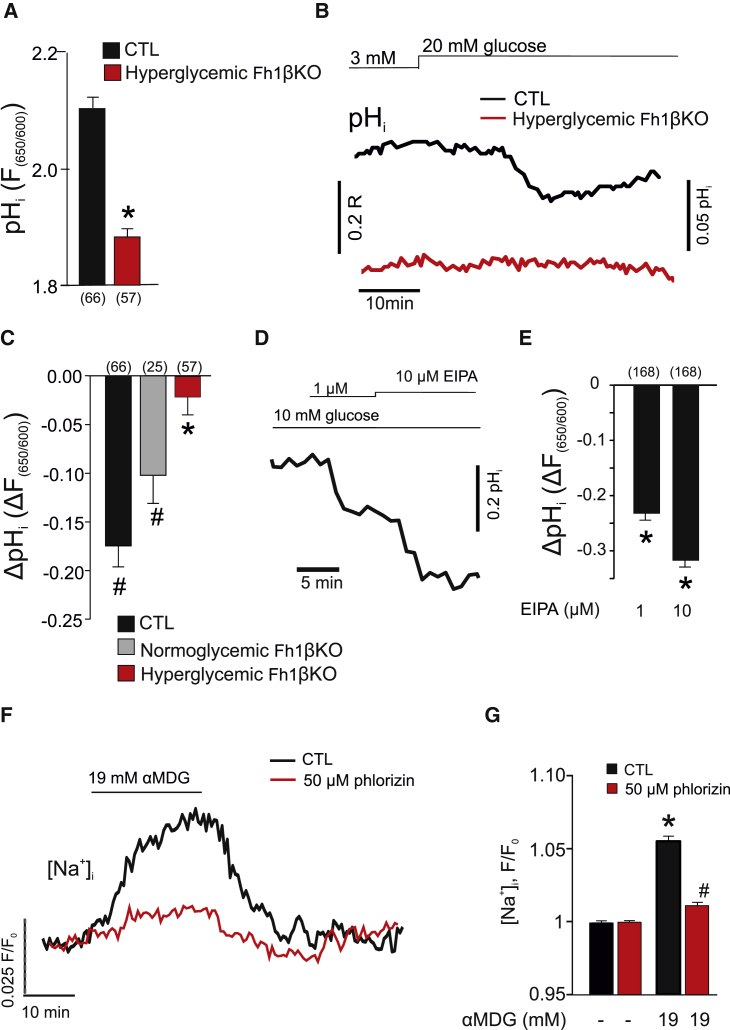
Hyperglycemia Results in Intracellular Acidification of α Cells (A) Histogram summarizing basal α cell intracellular pH (pH_i_) measured using the pH indicator seminaphthorhodafluor (SNARF) in α cells in islets from CTL (black) and hyperglycemic Fh1βKO (red) mice. Number of cells indicated below the respective bars in the histogram. ^∗^p < 0.05 versus CTL. (B) pH_i_ measured in α cells in islets from CTL (black) and hyperglycemic Fh1βKO (red) mice at 3 mM and 20 mM glucose. The traces have been offset to reflect the true difference in fluorescence ratios (*F*_650/550_) between CTL and Fh1βKO α cells. Measurements were performed in acutely isolated intact islets. (C) Net effect of 20 mM glucose on pH_i_ (Δ*F*_650/550_) in α cells from CTL (black) and nearly normoglycemic (gray) or hyperglycemic (red) Fh1βκO mice. Number of cells (n) indicated above the respective bars in the histogram. ^∗^p < 0.05 versus CTL; ^#^p < 0.05 versus basal (at 3 mM glucose). (D) Dose-dependent acidification of wild-type α cells in response to ethyl-isopropyl amiloride (EIPA) measured using the pH indicator SNARF. (E) Histogram summarizing the effect of EIPA on α cell pH_i_ in wild-type islets. Number of cells indicated above the respective bars in the histogram. ^∗^p < 0.05 versus basal (no EIPA). (F) Effect of the non-metabolizable glucose analogue α-methyl-D-glucopyranoside (αMDG) on cytoplasmic Na^+^ ([Na^+^]_i_) in wild-type α cells in the absence (black) and the presence (red) of phlorizin. Glucose (1 mM) was present throughout. [Na^+^]_i_ was measured using Sodium Green. Fluorescence (*F*) values have been normalized to that at 1 mM glucose (*F*_0_). (G) Effect of αMDG on [Na^+^]_i_ in the absence (black) and presence (red) of phlorizin in wild-type islets, as indicated. Data in (A) and (B) normalized to Sodium Green fluorescence under basal conditions (1 mM glucose; n = 59 cells) ^∗^p < 0.05 versus 1 mM glucose; ^#^p < 0.05 versus αMDG in the absence of phlorizin. Data are presented as mean values ±SEM of indicated number of experiments (n). In (B), (D), and (F), representative single-cell traces have been selected for display. See also Figures S2 and S3.

β cells from Fh1βKO mice have a lower cytoplasmic pH (pH_i_), an effect that was attributed to the accumulation of acidic fumarate (Adam et al., 2017), and pH_i_ is also lower in α cells from hyperglycemic Fh1βKO mice than in CTLs (Figure 4A). Although increasing glucose to 20 mM reduced pH_i_ in α cells in islets from both CTL and normoglycemic Fh1βKO mice, no acidification was seen in α cells from hyperglycemic Fh1βKO mice (Figures 4B and 4C).

### Intracellular Acidification Results from SGLT-Dependent Intracellular Na^+^ Accumulation

Intracellular pH is controlled by plasmalemmal and intracellular Na^+^-H^+^ exchangers (NHEs) (Casey et al., 2010). The significance of NHEs in the regulation of α cell pH_i_ is illustrated by the prompt reduction of pH_i_ observed when extracellular Na^+^ is reduced from the normal 140 mM to 10 mM (Figures S3A and S3B). Of the NHEs, NHE1 (*SLC9A1*) and NHE6 (*SLC9A6*) are expressed at particularly high levels in mouse and human α cells (Blodgett et al., 2015, DiGruccio et al., 2016). We used ethylisopropyl amiloride (EIPA), an inhibitor of plasmalemmal NHEs (Masereel et al., 2003), to explore the role of NHEs in control α cells where pH_i_ and normal ionic gradients are maintained. In wild-type islets exposed to 10 mM glucose, EIPA produced a concentration-dependent intracellular acidification in α cells (Figures 4D and 4E).

Glucose uptake in α cells is partially mediated by SGLTs (Bonner et al., 2015). We therefore speculated that hyperglycemia may cause intracellular acidification via an increase in the intracellular Na^+^ concentration ([Na^+^]_i_).

Although islets express both SGLT1 (encoded by *Slc5a1*) and SGLT2 (*Slc5a2*), SGLT1 is expressed at much higher levels than SGLT2 but still only at ∼1% of levels found in the kidney (Figure S3C). In mouse α cells, SGLT1 (*Slc5a1*) is expressed at very low levels (∼1%) compared with GLUT1 (*Slc2a1*) and GLUT3 (*Slc2a3*) (DiGruccio et al., 2016), and their contribution to glucose uptake is therefore likely to be negligible. Despite the low expression in mouse α cells, the SGLTs nevertheless result in glucose-dependent Na^+^ uptake. We demonstrated this using the non-metabolized SGLT substrate α-methyl-D-glucopyranoside (αMDG) (Wright et al., 2011). As shown in Figures 4F and 4G, application of αMDG increased [Na^+^]_i_ in α cells and this effect was almost fully prevented by the SGLT inhibitor phlorizin (Wright et al., 2011).

### SGLT-Mediated Na^+^ Uptake Leads to Intracellular Acidification and Reduced ATP Production in the Hyperglycemic α Cell

**Figure 5:**
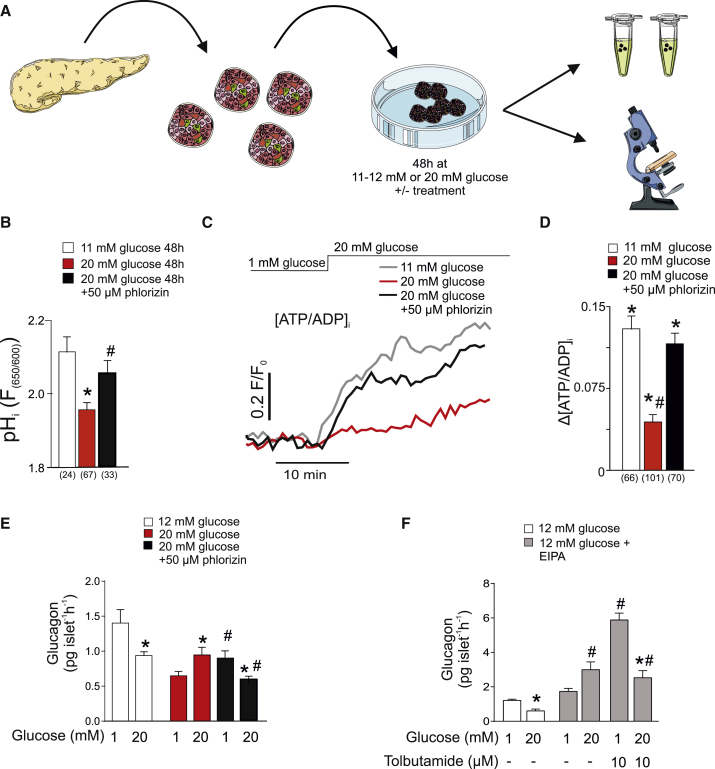
SGLT-Mediated Na^+^ Uptake Leads to Intracellular Acidification and Reduced ATP Production in the Hyperglycemic α Cell (A) Schematic of *ex vivo* experiments. Islets are isolated from mouse pancreas and incubated in 11–12 or 20 mM glucose for 48 hr. These concentrations approximate to fed plasma glucose levels before and after hyperglycemia develops in Fh1βKO mice (see Figures S1A and S1B). Figure was made using Servier medical ART. (B) Histogram summarizing basal pH_i_ measured using the pH indicator SNARF in α cells from wild-type islets incubated for 48 hr at 11 mM or 20 mM glucose, or 20 mM glucose plus 50 μM phlorizin. Number of cells (n) indicated below the respective bars in the histogram; ^∗^p < 0.05 versus 11 mM glucose. ^#^p < 0.05 versus 20 mM glucose. (C) Cytoplasmic ATP/ADP ratio at 1 and 20 mM glucose (indicated above recording) in α cells from wild-type islets cultured for 48 hr at 11 mM or 20 mM glucose, or 20 mM glucose plus 50 μM phlorizin. (D) Histogram summarizing the net effect of glucose on the cytoplasmic ATP/ADP ratio (ΔATP/ADP_c_) in α cells from wild-type islets cultured at 11 or 20 mM glucose in the absence or presence of 50 μM phlorizin as indicated. ^∗^p < 0.05 versus 1 mM glucose; ^#^p < 0.05 versus 20 mM glucose in islets cultured at 11 mM glucose. (E) Glucagon secretion measured at 1 or 20 mM glucose in wild-type islets cultured for 48 hr at 12 mM, 20 mM glucose, or 20 mM glucose plus 50 μM phlorizin as indicated. ^∗^p < 0.05 versus 1 mM glucose in respective group; ^#^p < 0.05 versus 20 mM glucose in islets cultured at 20 mM glucose in the absence of phlorizin (n = 8 experiments using islets from six mice). (F) As in (E) using wild-type islets that were cultured for 48 hr at 12 mM glucose alone (white bars) or in the presence of 10 μM NHE inhibitor EIPA (gray bars) in the absence or presence of 10 μM tolbutamide as indicated. ^∗^p < 0.05 versus 1 mM glucose in respective group; ^#^p < 0.05 versus 20 mM glucose in islets cultured in the absence of EIPA; ^#^p < 0.05 versus 1 mM glucose EIPA-incubated islets in the absence of tolbutamide (n = 3–8 experiments using islets from five mice). Data are presented as mean values ±SEM of indicated number of experiments (n). Traces in (C) represent average response in all cells. See also Figure S4.

We established a tissue culture protocol to test the effects of high glucose and Na^+^ uptake on α cell function. Briefly, isolated wild-type islets were cultured for 48 hr at 5, 11–12 (∼11), or 20 mM glucose (Figure 5A). We found that culturing islets at ∼11 mM glucose was optimal for both glucose-regulated glucagon and insulin secretion. Thus, we used ∼11 mM glucose as the “normoglycemic” CTL. This glucose concentration is in fact close to the fed plasma glucose levels in CTL mice (Figures S1A and S1B; see also https://phenome.jax.org/measures/32301) and similar to that found in Fh1βKO mice before hyperglycemia and the defects of glucagon secretion developed (Figures 1A, S1A, and S1B).

We confirmed that culture in high glucose (20 mM) for 24 hr resulted in 2SC staining of peripheral islet cells that was not observed when islets were cultured at 5 mM glucose (Figure S4A).

Culture for 48 hr at 20 mM glucose led to intracellular acidification of α cells compared with α cells in islets cultured at 11 mM glucose. Quantitatively, this effect was similar to the acidification observed in α cells in acutely isolated islets from hyperglycemic Fh1βKO mice compared with normoglycemic CTLs (compare Figures 4A and 5B). Importantly, the effect of high glucose culture was almost fully prevented when the SGLT inhibitor phlorizin was included in the culture medium (Figure 5B). “Hyperglycemia” *in vitro* did not result in acidification of the β cells but rather increased pH_i_ in these cells. This effect was not affected by phlorizin (Figure S5A), suggesting that different mechanisms control pH_i_ in α and β cells.

We investigated the impact of high glucose culture on the cytoplasmic ATP/ADP ratio (ATP/ADP_c_). A glucose-induced increase in ATP/ADP_c_ underlies the reduction of α cell K_ATP_ channel activity proposed to culminate in inhibition of glucagon secretion (see schematic in Figure S7). This would account for the reduced α cell [Ca^2+^]_i_ oscillatory activity in hyperglycemic Fh1βKO mice and the stimulation by high glucose/tolbutamide. In islets cultured at 11 mM glucose, raising the glucose concentration from 1 to 20 mM (after a pre-incubation period of ∼20 min at 1 mM glucose) led to an increase in ATP/ADP_c_ (Figures 5C and 5D). However, in islets cultured at 20 mM glucose, acutely elevating glucose from 1 to 20 mM produced a much smaller increase in ATP/ADP_c_. This effect of hyperglycemia was prevented in the presence of phlorizin and glucose remained capable of increasing ATP/ADP_c_ even when islets were cultured at 20 mM glucose with phlorizin.

Finally, we examined the impact of high glucose culture and intracellular acidification on glucagon secretion. Islets cultured at 12 mM glucose responded to an elevation of glucose from 1 to 20 mM glucose with inhibition of glucagon secretion (Figure 5E). However, following culture of islets at 20 mM glucose, glucagon secretion measured at 1 mM glucose was reduced by 50% compared with that seen in islets cultured at 12 mM glucose. Furthermore, increasing the glucose concentration stimulated rather than inhibited glucagon secretion (echoing what is observed in Fh1βKO islets from hyperglycemic mice; Figure S1D). Both these effects of high glucose culture were reversed by phlorizin. In contrast, the SGLT2 inhibitor dapagliflozin did not restore normal glucose regulation in high glucose-cultured islets (not shown), in agreement with the low expression of *Slc5a2* in mouse islets (Figure S3C).

The effects of high glucose incubation were also studied in human islets following culture at 5 or 20 mM glucose for 24 hr. Although islets cultured at 5 mM (normoglycemia in humans) responded to high glucose with 40%–60% suppression of glucagon secretion, no suppression of glucagon secretion by elevated glucose was detected in islets cultured at 20 mM glucose (Figure S4B).

In mouse islets cultured at 20 mM glucose for 48 hr, insulin secretion subsequently measured at 1 mM glucose was not affected, but the response to 20 mM glucose increased by >700% compared with islets cultured at 12 mM glucose (Figure S5B). Phlorizin did not affect insulin secretion (echoing the lack of effects on pH_i_), and it is consequently unlikely that the effects on glucagon secretion are secondary to changes of insulin secretion.

The capacity of phlorizin to counteract the adverse effects of chronic exposure to high glucose on glucagon secretion was not simply due to inhibition of α cell electrical activity: in α cells exposed to 20 mM glucose, there was no effect on the interspike membrane potential, the peak potential of the action potential, and action potential frequency (Figures S4C and S4D).

We considered whether intracellular acidification is enough to cause dysregulation of glucagon secretion. We incubated islets for 48 hr at 12 mM glucose in the absence or presence of the NHE inhibitor EIPA (10 μM). Although islets cultured at 12 mM glucose alone responded to a subsequent increase in glucose from 1 to 20 mM with inhibition of glucagon secretion, islets cultured in the presence of EIPA exhibited an inverted response and elevation of glucose stimulated glucagon secretion (Figure 5F), echoing what is seen in Fh1βKO islets and in wild-type islets after high glucose culture (Figures S1D and 5E, respectively). We examined whether this might be due to increased K_ATP_ channel activity. Indeed, treatment of islets with 10 μM tolbutamide increased glucagon secretion at 1 mM and restored normal glucose regulation of glucagon secretion by high glucose in EIPA-treated islets (Figure 5F). Effects similar to those produced by EIPA on glucagon secretion were obtained during long-term exposure to D-glyceraldehyde (Figure S4F), which also produces intracellular acidification (Figures S4D and S4E).

### Reduction in Hyperglycemia Rescues Glucagon Secretion, but Not Succination

**Figure 6:**
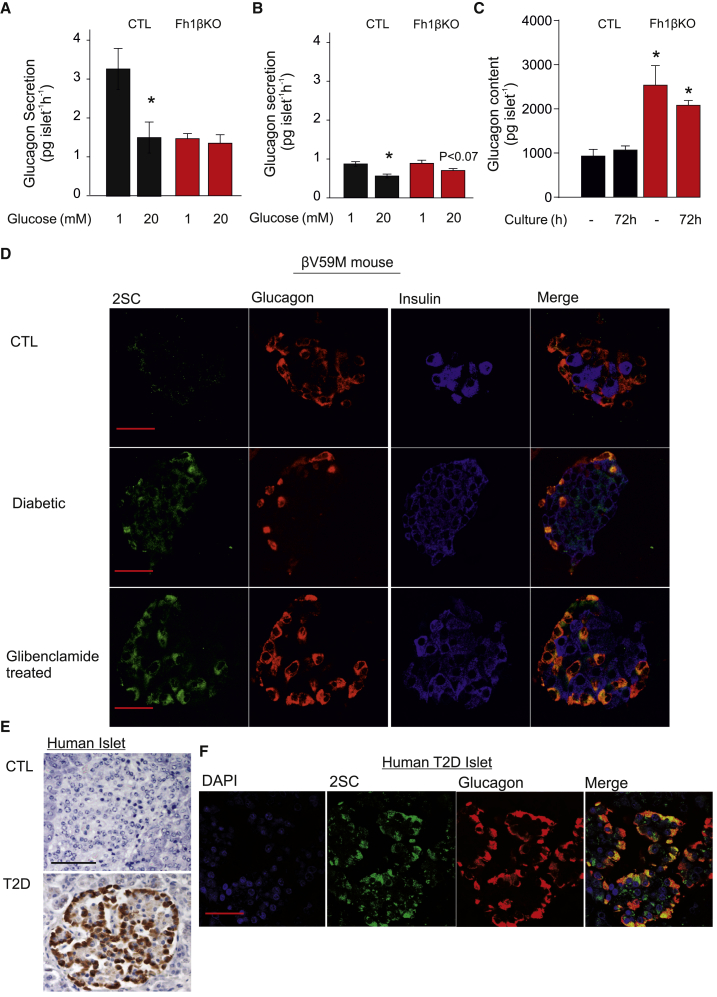
Protein Succination Persists after Restoration of Normoglycemia (A) Glucagon secretion at 1 and 20 mM glucose in acutely isolated islets from CTL and hyperglycemic Fh1βKO mice. ^∗^p < 0.05 versus 1 mM glucose (n = 9 experiments using islets from four mice of each genotype). (B) As in (A) but after 72 hr of culture at 12 mM glucose. ^∗^p < 0.05 versus 1 mM glucose (n = 9 experiments for each genotype using islets from four CTL and four Fh1βKO mice). (C) Glucagon content in CTL and Fh1βKO islets either acutely isolated or after 72 hr of culture. ^∗^p < 0.05 versus CTL. (D) Immunofluorescence for 2SC (green), glucagon (red), insulin (blue), and overlay (yellow) islets from CTL, hyperglycemic βV59M (diabetic), and normoglycemic βV59M mice (treated with glibenclamide). Note strong 2SC labeling of most glucagon-positive cells. Scale bar, 50 μm. (E) IHC for succination (2SC) in islets from non-diabetic (CTL) individuals and patients with type-2 diabetes (T2D). Scale bar, 50 μm. (F) Immunofluorescence for 2SC (green), glucagon (red), DAPI (blue), and overlay (yellow) in islets from patients diagnosed with T2D. Note strong 2SC labeling of most glucagon-positive cells. Scale bar, 50 μm. Data in (E) and (F) are representative of six donors for both the non-diabetic (CTL) and T2D groups. Data are presented as mean values ± SEM of indicated number of experiments (n). See Table S1 for details on the donors.

If the dysregulation of glucagon secretion in diabetic Fh1βKO is caused by hyperglycemia, then it should be possible to reverse the secretion defect simply by culturing the islets at normal glucose. Glucagon secretion in acutely isolated islets from hyperglycemic Fh1βKO mice was only ∼40% of that in CTL islets and was unaffected by glucose, unlike what was seen in CTL islets (Figure 6A). Culture alone reduced glucagon secretion in CTL islets by >70% compared with acutely isolated islets, but residual glucagon secretion remained inhibited when glucose was increased from 1 to 20 mM (Figure 6B). There was a tendency toward restoration of normal glucose regulation when Fh1βKO islets from hyperglycemic mice were cultured at 12 mM glucose for 72 hr (Figure 6B). Islets from Fh1βKO mice contain 200% more glucagon than non-diabetic CTLs, a difference that was only partially reversed after culturing the islets at 12 mM glucose (Figure 6C).

The dysregulation of glucagon secretion in βV59M mice is associated with strong 2SC labeling of the α cell (Figure 3A). We used this mouse model to test the reversibility of protein succination (i.e., without genetic deletion of *Fh1* and strong 2SC labeling of the β cells). In normoglycemic βV59M mice (i.e., before tamoxifen treatment), no succination was seen in islet cells (Brereton et al., 2014). Following transgene induction and 14 days of hyperglycemia, stronger 2SC staining was observed in the α cells than β cells. Normoglycemia was then restored by treatment with glibenclamide. However, even after 2 weeks of glibenclamide treatment, the α cells remained 2SC positive (Figure 6D).

Persistent 2SC labeling of peripheral cells (likely to be α cells) was also seen in islets from diabetic GK rats 10 days after plasma glucose levels had been normalized by bariatric (Roux-en-Y gastric bypass [RYGB]) surgery (Ramracheya et al., 2016); no 2SC labeling was observed in normoglycemic control (Wistar) rats (Figure S6A). Despite the normalization of plasma glucose, glucagon secretion at 1 mM glucose remained low in RYGB rats and 6 mM glucose was without a statistically significant inhibitory effect (Figure S6B).

Protein succination (2SC staining) was also seen in postmortem specimens of islets/pancreases from T2D patients and absent in islets from non-diabetic individuals (Figure 6E). In human T2D pancreatic islets (Table S1), 2SC staining appeared to be most prominent in α cells (Figure 6F).

### Protein Succination Is Observed in Cardiomyocytes and Renal Tubular Cells from Hyperglycemic Fh1βKO Mice

**Figure 7:**
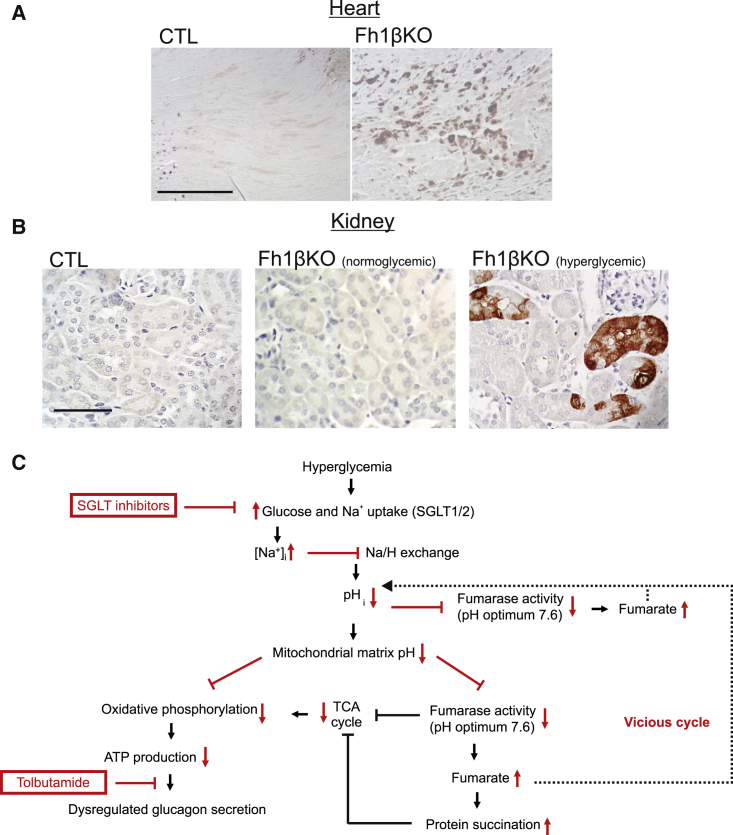
Protein Succination in Renal Tubular Cells and Cardiomyocytes from Hyperglycemic Fh1βKO Mice (A) IHC for 2SC in CTL (left) and diabetic Fh1βKO (right) hearts showing staining in a subset of cells in diabetic animals but not in the non-diabetic CTLs. Scale bar, 50 μm. (B) As in (A) but showing sections of kidney in CTL (left) and normoglycemic (middle) or hyperglycemic (right) Fh1βKO mice as indicated. (C) Schematic of the proposed relationship between hyperglycemia, impaired ATP production, and protein succination. The increased intracellular fumarate resulting from reduced activity of fumarase leads to protein succination. The sites of action of SGLT inhibitors and tolbutamide are indicated. Sections in (A) and (B) are representative of observations in >10 animals of both genotypes.

Evidence for protein succination (2SC staining) was also observed in cardiomyocytes (Figure 7A) and renal tubular cells (Figure 7B) in hyperglycemic Fh1βKO mice, but not in non-diabetic littermate CTLs or young Fh1βKO mice. However, 2SC staining was scattered, suggesting that fumarate levels were only elevated in some cells.

2SC labeling of α cells and renal tubular cells was not reversed by administration of phlorizin *in vivo* (400 mg/kg subcutaneously) to hyperglycemic Fh1βKO mice (plasma glucose: >20 mM) for 7 days (data not shown). This is consistent with the persistence of 2SC labeling in the GK rats and βV59M mice following normalization of plasma glucose.

## Discussion

We demonstrate that chronic hyperglycemia results in changes in glucagon secretion similar to those that occur in diabetes. These changes, which occur in three hyperglycemic rodent models of diabetes, manifest as a reduction of glucagon secretion at low glucose and stimulation, rather than inhibition, in response to high glucose. Similar disturbances are induced after culture of mouse and human islets under hyperglycemic conditions (20 mM glucose for 24–48 hr). Our findings are broadly consistent with the report that chronic glucose infusion in rats leads to moderate hyperglycemia (∼15 mM) and hyperglucagonemia, despite stimulation of insulin secretion (Jamison et al., 2011).

### Intracellular Acidification Links Hyperglycemia to Impaired ATP Production

We propose a model that explains the dysregulation of glucagon secretion induced by hyperglycemia (Figure 7C). In this model, hyperglycemia results in increased Na^+^ uptake mediated by plasmalemmal SGLTs and a consequential increase in [Na^+^]_i_. The transmembrane Na^+^ gradient normally drives the uphill (i.e., against an electrochemical gradient) transport of several molecules in the cell, including H^+^. An increase in [Na^+^]_i_ will therefore interfere with the removal of H^+^ from the cytoplasm. This will cause intracellular acidification (i.e., an increased intracellular H^+^ concentration).

The critical role played by intracellular acidification as the causal factor leading to dysregulation of glucagon secretion is underscored by the effects of EIPA. Addition of this NHE inhibitor leads to acidification and long-term treatment results in inverted glucose regulation of glucagon secretion (i.e., stimulation rather than inhibition) that can be corrected by low concentrations of tolbutamide.

In astrocytes, mild cytoplasmic acidification results in a marked reduction of intramitochondrial (matrix) pH (pH_m_) and inhibition of oxidative metabolism (Azarias et al., 2011). By analogy, we propose that the reduction of pH_i_ and pH_m_ induced by hyperglycemia will reduce the activity of both the cytoplasmic and mitochondrial forms of fumarase in α cells, accounting for the increased 2SC labeling. Because fumarate is an acid, reduced fumarase activity (via accumulation of fumarate) will result in further acidification, thus establishing a vicious cycle of progressive intracellular acidification. Fumarate can leave the mitochondria and be processed in the cytosol, as shown by the observation that the cytoplasmic form of fumarase (lacking the mitochondrial leading sequence) when re-introduced into *Fh1*-deficient cells “rescues” many of the consequences of deletion of *Fh1* (Adam et al., 2013, Adam et al., 2017, Ternette et al., 2013). We speculate that reverse operation of this mechanism could result in transport of cytoplasmic fumarate into the mitochondria and thus contribute to matrix acidification.

The metabolic consequences of reduced fumarase activity may be exacerbated by the effects of fumarate on other metabolic enzymes. For example, fumarate reduces the activity of other enzymes integral to both glycolysis and the TCA cycle, including glyceraldehyde-3-phosphate dehydrogenase (GAPDH) and aconitase (Blatnik et al., 2008, Ternette et al., 2013).

An alkaline pH_m_ is required for ATP production, which is energized by the downhill movement of H^+^ in an electrochemical gradient across the inner mitochondrial membrane. In β cells, alkalinization of the mitochondrial matrix has been shown to correlate with mitochondrial ATP production (Akhmedov et al., 2010). When the mitochondrial matrix has undergone sufficient acidification, H^+^ flux through ATP synthase becomes sufficiently reduced to impair ATP production.

The resultant fall in the ATP/ADP ratio results in increased K_ATP_ channel activity. Closure of the K_ATP_ channels is required for normal glucose regulation of glucagon secretion. This model explains why the effects of chronic hyperglycemia and inhibition of NHEs resemble those produced by inhibition of mitochondrial ATP synthesis by oligomycin or pharmacological activation of the K_ATP_ channels by diazoxide (Zhang et al., 2013). We found that a low concentration of the K_ATP_ channel inhibitor tolbutamide increased glucagon secretion at 1 mM glucose and restored normal glucose regulation (inhibition by high glucose) in islets from hyperglycemic Fh1βKO and βV5M mice, whereas it reduced glucagon secretion at low glucose in CTL mice. These observations are consistent with a bell-shaped relationship between K_ATP_ channel activity and glucagon secretion (see legend to Figure S7) and is also supported by our observation of reduced [Ca^2+^]_i_ oscillatory activity in α cells from diabetic Fh1βKO mice (Figures 3F and 3G).

Phlorizin also restores glucose-regulated glucagon secretion, but its mode of action is completely different to that of tolbutamide. Although tolbutamide corrects the “symptoms” of the defect, phlorizin corrects the cause. By inhibition of SGLT activity, phlorizin prevents Na^+^ uptake and intracellular acidification with resultant preservation of mitochondrial ATP production.

### Changes in Glucagon Content with Hyperglycemia and Loss of FH

Although the effects on glucagon secretion of deleting *Fh1* in α and β cells are similar, the effects on glucagon content are not. Thus, although glucagon content is increased in Fh1βKO islets, it is decreased in Fh1αKO islets. The reduction of glucagon content in the Fh1αKO mice might be explained by the activation of cataplerosis to replenish the Krebs cycle intermediates following the complete genetic deletion of fumarase. Deamination of the amino acids that would otherwise be used for glucagon biosynthesis represents an obvious source, similar to the explanation for the >95% reduction of insulin content in β cells from Fh1βKO mice (Adam et al., 2017). The strong reduction of glucagon content (∼90%) is probably a major factor explaining the 90% decrease in glucagon secretion in Fh1αKO islets. Importantly, in Fh1αKO islets, glucose tends to stimulate rather than inhibit glucagon secretion, echoing what is seen in the presence of the membrane-permeable exogenous dimethyl fumarate and in islets isolated from hyperglycemic mice. It is unclear precisely why glucagon content is increased in hyperglycemic Fh1βKO islets and in islets from human T2D donors. The simplest explanation is that the rate of secretion is reduced by 70%. Although fumarase activity is reduced sufficiently to result in 2SC staining, the α cells in the hyperglycemic models may nevertheless retain sufficient enzyme activity not to deplete the amino acid pool needed for glucagon biosynthesis.

### Functional Significance of Protein Succination

It is interesting that α cells in islets from patients with T2D showed strong protein succination. We also observed particularly strong 2SC labeling of α cells in diabetic βV59M mice, whereas staining of the β cells was much weaker. Thus, the impact of hyperglycemia in α and β cells must be quite different and it is interesting that chronic hyperglycemia has opposite effects in α (acidification) and β cells (alkalinization). Moreover, phlorizin corrected the dysregulation of glucagon secretion resulting from chronic hyperglycemia but did not affect the hypersecretion of insulin.

We also found that protein succination was poorly reversible; even after 2 weeks of glibenclamide treatment and normoglycemia, α cells in βV59M showed protein succination. In this context it is worth remembering that protein succination is an irreversible chemical reaction. Thus, the lifespan of the succinated proteins must exceed 2 weeks. It is also possible that succination interferes with protein degradation and/or that hyperglycemia results in permanent dysregulation of metabolism as a consequence of hyperglycemic stress. The identity of all the succinated proteins remains unknown, but immunohistochemistry suggests it is extranuclear. Previous analyses of mouse and human islets indicate that mitochondrial proteins like DJ-1 (a cellular anti-oxidant response regulator) (Eberhard and Lammert, 2017, Jain et al., 2012) become succinated in hyperglycemic Fh1βKO islets and in diabetic human islets (Adam et al., 2017). The slow reversibility of succination may also explain why restoration of normoglycemia (by culture or by RYGB surgery) is associated with only partial recovery of normal glucose regulation of glucagon secretion.

### Protein Succination and Reduced Cardiac Mortality and Renal Failure in Diabetic Patients Treated with SGLT2 Inhibitors

Interestingly, protein succination was also observed in renal cells and cardiomyocytes, two cell types affected by secondary complications of diabetes. This suggests that that the model can be extended to other SGLT-expressing cells and that hyperglycemia also leads to inhibition of fumarase in these cells. As in α cells, reduced fumarase activity and intracellular acidification can be expected to compromise ATP production and thereby predispose to renal (Forbes, 2016) and cardiac failure (Brown et al., 2016). This model is supported by the recent observation that inhibition of SGLTs leads to reduced Na^+^ transport in the heart (Bertero et al., 2018). We emphasize that there is no increase in plasma fumarate levels in Fh1βKO mice. Thus, we can exclude any possibility that export of fumarate from the β cells may account for the 2SC labeling in the heart (which is unlikely also from quantitative considerations).

We emphasize that protein succination should be regarded as a biomarker of impaired mitochondrial metabolism rather than the cause. Given that both heart and kidneys express SGLT2 (although at 10-fold higher levels in kidneys), this model may give some insight into the dramatic reduction in cardiac mortality and kidney disease observed in diabetic patients treated with SGLT2 inhibitors (Wanner et al., 2016, Zinman et al., 2015). Explanations to date have focused on altered intermediary metabolism (Ferrannini, 2017, Lopaschuk and Verma, 2016). The “Na^+^ toxicity” hypothesis outlined here not only provides an alternative mode of action but also suggests a unifying molecular mechanism underlying the spectrum of diabetes-associated co-morbidities. The hypothesis we propose posits that tissues that express SGLTs will be particularly sensitive to hyperglycemia.

### Limitations of Study

The concepts presented in these studies are based on animal experiments and it is now important to extend the model to human islets (and especially those from diabetic patients). Ultimately, it will also be important to translate these observations into improved therapy for diabetic patients. This will involve the demonstration that normal counter-regulatory glucagon secretion can be restored in diabetic patients by treatment with SGLT inhibitors or low-dose sulfonylurea. In addition, we acknowledge that, although protein succination, similar to that found in α cells, is also observed in renal tubular cells and cardiac myocytes, the upstream molecular mechanism leading to reduced fumarase activity in these cells may be distinct from that in α cells.

## STAR★Methods

### Key Resources Table

**Table:** 

REAGENT or RESOURCE	SOURCE	IDENTIFIER
**Antibodies**		

Guinea pig anti-Insulin	in-house	N/A
Mouse polyclonal anti-Glucagon	Sigma	G2654; RRID: AB_2313773
Rabbit anti-2SC	N/A	Prof. N Frizzell
Rabbit anti-porcine FH	Autogen Bioclear or Nordic Immunology	NEO54

**Bacterial and Virus Strains**		

Perceval	Berg et al., 2009	Addgene Plasmid #21737

**Biological Samples**		

Human Islets	Diabetes Research & Wellness Foundation Human Islet Isolation unit	Oxford, UK

**Chemicals, Peptides, and Recombinant Proteins**		

Phlorizin	Cayman	11576
Tolbutamide	Sigma	T0891
EIPA (Ethylisopropyl amiloride)	Tocris Bioscience	Cat. No. 3378
αMDG (α-methyl-D-glucopyranoside)	Sigma Aldrich	M 9376

**Critical Commercial Assays**		

Glucagon EURIA	Euro diagnostica	RB310
RAT - Insulin RIA	Millipore	RIK-13
MSD Mouse/Rat Insulin, Glucagon Kit	Mesoscale discovery	K15145C

**Experimental Models: Cell Lines**		

αTC1-6 cell line	ATACC	CRL-2934; RRID: CVCL_8036

**Experimental Models: Organisms/Strains**		

Tg(*Ins2-Cre*)^*23Herr*^Cre recombinase, *Rip2-Cre*^*+/-*^	Herrera, 2000	N/A
C57BL/6J mice	Jackson laboratories	000664 -C57BL/6J
*Glu*-i*Cre*^+/-^	Parker et al., 2012	N/A
NMRI	Jackson laboratories	009682 - NMRI-Tbce<pmn>/J
Goto-Kakizaki (*TohiCskCrljCr*) GK rats	Ramracheya et al., 2016	N/A
*Fh1*^*tm1Pjpfl/fl*^	Pollard et al., 2007	N/A
βKir6.2-V59M mice	Brereton et al., 2014	N/A

**Software and Algorithms**		

IGOR Pro	Wavemetrics	https://www.wavemetrics.com/downloads/current
Fiji	ImageJ	https://imagej.net/Fiji/Downloads

**Other**		

SNARF-5F 5-(and-6)-Carboxylic Acid	Thermo Fisher	S23922
Sodium Green Tetraacetate, cell permeant - Special Packaging	Thermo Fisher	S6901
Fluo-4, AM, cell permeant	Thermo Fisher	F14201

### Contact for Reagent and Resource Sharing

Further information and requests for resources and reagents should be directed to and will be fulfilled by the Lead Contact, Patrik Rorsman (patrik.rorsman@drl.ox.ac.uk).

### Experimental Model and Subject Details

#### Animal Models: Mice

All animal experiments were conducted in accordance with the UK Animals Scientific Procedures Act (1986) and University of Oxford local ethical guidelines. Animals were kept on a 12 hr light: dark cycle, at 22°C. The mice used were either *Fh1*^*tm1Pjpfl/fl*^*Rip2-Cre*^*+/-*^ (designated Fh1βKO), which were generated originally by crossing *Fh1*^*tm1Pjpfl/fl*^ (Pollard et al., 2007) with Tg(*Ins2-Cre*)^*23Herr*^Cre recombinase, *Rip2-Cre*^*+/-*^ (Herrera, 2000) (as described in Adam et al., 2017), or *Fh1*^*tm1Pjpfl/fl*^*Glu*-i*Cre*^+/-^ (designated Fh1αKO), which were generated by crossing *Fh1*^*tm1Pjpfl/fl*^ (Pollard et al., 2007) with *Glu*-i*Cre*^+/-^ (Parker et al., 2012). Mice had been backcrossed on a C57BL/6J background at least 5 times when the experiments commenced. *Fh1* was deleted specifically in either β- or α-cells respectively in these strains and littermates were used as controls.

Details of βKir6.2-V59M mice (that express a gain-of-function K_ATP_ channel mutation in the β-cells following tamoxifen induction) are as described previously (Brereton et al., 2014). These mice were generated originally by crossing Kir6.2-V59M (Girard et al., 2009) with mice expressing a tamoxifen inducible rat insulin promoter II (RIPII-Cre-ERT mice) (Dor et al., 2004). Mice were on a mixed (C3H, C57BL/6, 129/sv) genetic background. Kir6.2-V59M expression was induced in pancreatic β-cells in mice at 12-14 weeks of age by a single subcutaneous injection of 0.4 ml of 20 mg/ ml tamoxifen in corn oil (Sigma). Littermates were used as controls.

Some experiments were carried out with islets isolated from NMRI or C57BL/6J mice (referred to as wild-type) obtained from a commercial supplier.

#### Animal Models: Rats

Experiments on rats were performed as described previously (Ramracheya et al., 2016). Briefly, adult male Wistar and Goto-Kakizaki (*TohiCskCrljCr*) GK rats were used. The GK rats were divided into either Roux-Y-gastric bypass procedure (RYGB) or sham-operation groups.

Animals of both sexes were used for all islet experiments; neither have we observed nor has it been reported that there are sex differences at the islet level. Therefore, we found it best to include both sexes, and in these studies we have not observed any sex differences. We have previously reported, and discussed sex differences in the FH1βKO mouse model (Adam et al., 2017). For all other *in vivo* experiments male mice were used.

### Method Details

#### Islet Isolation, Islet Culture and Hormone Secretion from Mouse Islets

Islets were isolated by collagenase or liberase digestion. After isolation, islets were transferred to RPMI-1640 supplemented with 5 mM glucose, 100 U/ml penicillin, 10 μg/ml streptomycin (P/S) and 10% fetal calf serum (FCS). In most experiments they were kept in this medium at 37°C in a humidified atmosphere (5% CO_2_/95% air) for <2 hr prior to experiments. However, in some experiments, islets were cultured in RPMI-1640 (supplemented with 10% FCS and 1% P/S) containing either 5, 11-12 or 20 mM glucose, or 20 mM glucose to which was added either 50 μM of the SGLT inhibitor phlorizin (dissolved in DMSO; final concentration: 0.1% v/v), or the NHE inhibitor ethylisopropyl amiloride (EIPA, dissolved in DMSO: 0.1% v/v) for 48 hr prior to the hormone release measurements (as indicated). Dimethyl fumarate was dissolved in DMSO (0.1% v/v). Control experiments were performed in the presence of the same concentration of DMSO.

In all cases, hormone secretion was measured from batches of 10-12 islets. Size-matched islets were hand-picked and washed twice in glucose-free RPMI-1640 (supplemented with 100 U/ml penicillin, 10 μg/ml streptomycin and 10% FCS). They were pre-incubated for 1 hr in a humidified chamber at 37°C (5% CO_2_/95% air) in 300 μl of Krebs-Ringer buffer (KRB) which contained the following (mM) 140 NaCl, 3.6 KCl, 2.6 CaCl_2_, 0.5 MgSO_4_.7H_2_O, 0.5 NaH_2_PO_4_, 2 NaHCO_3_, 5 HEPES and 2 mg/ml BSA (pH adjusted to 7.4 with 1 M NaOH) and 1 mM glucose. The pre-incubation buffer was discarded and the islets were stimulated for a further 1 h with the test conditions indicated. An aliquot of the supernatant was collected and stored at -20°C for quantification of either insulin or glucagon secretion by radioimmunoassay. The remaining supernatant was discarded and the islets were lysed in 100 μl of ice-cold acid ethanol solution (containing ethanol, H_2_O and HCl in a ratio of 52:17:1) to release their hormone content. Lysates were immediately frozen at -20°C for later analysis. Insulin (Millipore) and glucagon (Euro-diagnostica) was determined by commercial radio-immunoassays following the manufacturer’s protocols.

#### Plasma Glucose Measurements

Fed blood glucose levels were determined with an Accuchek Aviva meter at approximately 9 am.

#### Plasma Fumarate Measurements

Fumarate was extracted from mouse plasma and analyzed as described previously (Spegel et al., 2013). Plasma fumarate concentrations were calibrated against an external standard of Na_2_-fumarate.

#### Glucose and Insulin Tolerance Test in Fh1αKO Mice

In the glucose tolerance tests, male Fh1αKO mice and control littermates were fasted for 6 hr from 8.30 am and fasting blood glucose was measured, the mice were then injected intraperitoneally with 2 g/kg body weight of D-glucose in PBS and blood glucose levels were measured 15, 30, 60 and 120 min post injection.

In the insulin tolerance tests, male Fh1αKO mice and control littermates were fasted for 3-4 hr prior to the experiments. Fast-acting human insulin (0.75 U/kg Actrapid, Novo Nordisk) was injected intraperitoneally with a 25gauge needle at time zero and samples taken for plasma glucose measurements after 15, 30, 60 and 120 min post injection.

#### Imaging of Cytosolic ATP/ADP, Ca^2+^, Na^+^ and pH in Mouse α-Cells

For these experiments, the extracellular medium consisted of (mM) 140 NaCl, 4.6 KCl, 2.6 CaCl_2_, 1.2 MgCl_2_, 1 NaH_2_PO_4_, 5 NaHCO_3_, 10 HEPES, (pH 7.4, with NaOH) and (unless otherwise stated) 1 glucose. When extracellular Na^+^ was lowered to 10 mM, NaCl was equimolarly replaced by N-methyl-D-glucamine. For measurements of [Na^+^]_i_, the membrane potential was held at ≈-70 mV by including the K_ATP_ channel activator diazoxide (0.2 mM) in the superfusion medium. The bath was perifused at 60-200 μl/min and the temperature kept at ∼34°C.

Time-lapse imaging of ATP/ADP ratio in islets was performed using a Zeiss AxioZoom.V16 zoom microscope, and magnifications between 10x and 14x. Mouse islets were transduced with an adenovirus (3×10^4^ infectious units per islet) delivering Perceval, a recombinant sensor of ATP/ADP based on circularly permutated YFP variant Venus (Berg et al., 2009). Perceval is pH-sensitive but the changes in pH_i_ account for <10% of the glucose-induced changes in Perceval fluorescence (Tarasov et al., 2012). Groups of islets isolated from littermate control and Fh1βKO animals were imaged simultaneously 24 hr post-infection, with single-cell resolution. Time-lapse images were collected every 30 s. The α-cells were identified by the ability of adrenaline to increase cAMP in cells infected with ‘red downward cADDis’ cAMP recombinant sensor delivered using a BacMam vector.

Parallel time-lapse imaging of [Ca^2+^]_i_ and pH_i_ in mouse islets was performed on an inverted Zeiss AxioVert 200 microscope equipped with Zeiss 510-META laser confocal scanning system, using 40x/1.3 objective. Cells that generate spontaneous [Ca^2+^]_i_ oscillations at low glucose were taken to represent α-cells. Mouse islets were loaded with 6 μM of the Ca^2+^ sensitive dye Fluo-4 for 90 min before being transferred to a separate solution containing 6 μM of the pH-sensitive dye SNARF-5F for a further 50 min at room temperature and imaged using an open chamber at 34°C. The ratiometric dye SNARF-5F was excited at 543 nm and emission was collected at 650 nm and 600 nm, which corresponds to the emission maxima of the sensor at pH 9 and pH 6, respectively. Fluo-4 was excited at 488 nm and imaged at 530 nm. Images were collected at the frequency of 0.03 Hz. The absolute pH_i_ change in Figure 4 was estimated using a high K^+^ (140 mM)-nigericin (10 μM)/valinomycin (5 μM) calibration protocol (Tarasov et al., 2012).

Sodium Green time-lapse measurements of [Na^+^]_i_ were performed in dispersed islet cells, on a Zeiss AxioZoom.V16 zoom microscope, using magnifications in the range 15x-20x. α-cells were distinguished by the positive cAMP response to 10 μM adrenaline (Vieira et al., 2004) (as described above). Islets were dispersed into a single-cell suspension, which was plated on 0.17 mm thick glass coverslips in 10 μl droplets and left to attach for >2 hr. Multiple droplets, consisting of cells of different genotypes, were plated on the same coverslip and imaged simultaneously; thus enhancing the statistical power of comparisons. After 24 hr, cells were pre-loaded with 6 μM of Sodium Green for 30 min and imaged at several locations throughout the coverslip simultaneously. Sodium Green was excited at 490 nm and emission was collected at 515 nm. Red downward cADDis was excited at 572 nm and the emission was collected at 629 nm. Image sequences were analyzed (registration, background subtraction, ROI intensity *vs* time analysis) using open-source FIJI software (http://fiji.sc/Fiji).

Singular time-lapse recordings of [Ca^2+^]_i_ were performed in intact freshly isolated mouse islets loaded with 6 μM Fluo-4 at room temperature (Molecular Probes) for 90 min and imaged using a Zeiss AxioVert 200 microscope equipped with Zeiss 510-META laser confocal scanning system, using 40x/1.3 objective. A 488 nm argon laser (3% intensity) was used to excite Fluo-4 and emission was collected at 530 nm, using 512x512 frame scanning mode with a pixel dwell time of 6 μs and a bit depth of 8-bit. Images were acquired every 3.93 s (∼0.25 Hz). α-Cells were identified by an increase in [Ca^2+^]_i_ in response to adrenaline (5 μM) (Hamilton et al., 2018). For the singular [Ca^2+^]_i_ recordings, the numerical data was analysed using IgorPro package (Wavemetrics), each trace was normalised (F/F_o_) and baseline/bleach corrected. Partial area under the curve (pAUC) was then calculated by splitting each trace into 30 s intervals and computing AUC for each interval using trapezoidal integration. pAUC points falling within a given region (e.g. 1 mM glucose) for each trace were averaged, the mean of these values across the α-cell population was then calculated to generate the final pAUC value (displayed in the column plot). Statistical analysis was performed using SPSS. Mann-Whitney U-test or Wilcoxon’s paired test were used to compute the significance of difference between independent and dependent samples, respectively. Differences with p < 0.05 were considered significant.

#### Immunohistochemistry and Immunofluorescence: Mouse, Rat and Human Pancreatic Tissue

Mouse and human pancreases were fixed in 10% neutral-buffered formalin, dehydrated and processed for paraffin wax embedding and sectioning (3 μm). Immunohistochemistry (IHC) was carried out using the EnVision kit (Dako) as per the manufacturer’s protocol with the following antibodies: FH, insulin, glucagon and 2SC (Nagai et al., 2007).

Immunofluorescence was performed using the same antibodies as for IHC with Alexa Fluor^®^ secondary antibodies using a Zeiss LSM510 META confocal imaging system.

Both staining and analysis of murine and human pancreatic sections were conducted blinded. Sections from human subjects were scored by 3 independent observers.

#### Fumarase Activity Measurements

Fumarase activity was determined by measuring the rate of NADH production from the coupled reaction converting fumarate to oxaloacetate *via* malate dehydrogenase (MDH) as described previously (Massey, 1953). pH-dependent changes in fumarase were measured on 0.05 U of porcine fumarase by adjusting the pH of the HEPES buffer using KOH. A 5 μl sample was loaded in a 96-well plate, 195 μl assay buffer (50 mM Hepes-KOH, 1 mM KH_2_PO_4_, 1 mM MgCl_2_, 10 mM NAD^+^, 10 mM L-glutamate, 6.75 U malate dehydrogenase, 2.5 U glutamate-oxaloacetate transaminase) was added and the plate was incubated for 10 min at 37°C. Following the incubation, 10 μl of 30 mM fumarate (Sigma) was added and the appearance of NADH was measured every 30 s at 37°C by excitation/emission at 360/450 nm (Enspire, 2300 Multilabel Reader, Perkin Elmer). NADH concentration was determined from a standard curve. All samples were run in triplicate and normalized to protein content, as measured by bicinchoninic acid protein assay.

#### Sglt Expression Analysis

Total RNA from mouse tissues was isolated using a combination of TRIzol and PureLink RNA Mini Kit. DNase treatment was included to eliminate DNA contamination. cDNA was synthesized using the High Capacity RNA-to-cDNA kit. Real-time qPCR was performed using SYBR Green detection and gene specific QuantiTect Primer Assay. Relative expression was calculated as 2^-ΔΔ*CT*^. *Actb* and *Ppia* were used for normalization.

#### Electrophysiological Measurements

Electrical activity measurements were conducted as previously described (Zhang et al., 2013). Briefly, membrane potential of islet cells was monitored in islet cells within freshly isolated intact mouse islets using perforated patch whole-cell technique carried out at 32–34°C. The recording was performed using an EPC-10 patch-clamping amplifier (HEKA Electronics, Lambrecht/Pfalz, Germany) and Pulse (version 8.80) software. Patch pipettes were pulled from borosilicate glass with the resistances of ∼5 MW when filled with the pipette solutions. The pipette solution contains (mM): 76 K_2_SO_4_, 10 NaCl, 10 KCl, 1 MgCl_2_ and 5 HEPES (pH 7.35 with KOH) and the extracellular solution consists of (mM): 140 NaCl, 3.6 KCl, 0.5 MgSO_4_, 1.5 CaCl_2_, 0.5 NaH_2_PO_4_, 5 NaHCO_3_ and 10 HEPES (pH 7.4 with NaOH). Glucose concentrations are as indicated in the figure. Amphotericin B (0.24 mg/ml) was included in the pipette solution for membrane perforation. Data analysis was performed using ClampFit (Version 9.2.0.11, Molecular Devices).

#### Human Islets and Ethics

Human pancreatic islets were isolated, with ethical approval and clinical consent, at the Diabetes Research and Wellness Foundation Human islet Isolation Facility (Oxford). For histology, pancreatic tissue blocks from the Oxford Human Pancreas Repository were used (licensed by the Human Tissue Authority).

#### Hormone Secretion from Human Islets

Human islets from three donors (2 female and 1 male; age: 52.33±0.8; BMI: 30±2; HbA1c: 5.6-5.7%) were cultured in RPMI-1640 (supplemented with 10% FCS and 1% penicillin and streptomycin) containing 5 mM glucose. Secretion experiments were performed as described above.

### Quantification and Statistical Analysis

All data are presented as mean ± standard error of mean (SEM) of the indicated number of experiments (n). All statistical tests were conducted in Prism5 (GraphPad Software, San Diego, CA). For two groupings, a t-test was conducted with the appropriate *post-hoc* test. For more than two groupings, a one-way ANOVA was performed. If the data passed normality criteria (D’Agostino’s test of normality and Bartlett’s test of equal variances) a parametric test was conducted with the appropriate *post-hoc* test (Student Newman-Keuls). If the normality criteria were not met, a Kruskal–Wallis test with Dunn’s multiple comparison test was conducted. Levels of significance are given in the figures for the indicated comparisons.

For analysis of imaging data, statistical significance of the differences between paired or unpaired samples were tested using Friedman or Kruskall-Wallis tests, respectively, with Nemenyi *post-hoc* analysis, as implemented in R package (R Development Core Team, 2016).

## Supplemental Information

Document S1. Figures S1–S7 and Table S1

Document S2. Article plus Supplemental Information
