# Supplementary material for: Dysregulation of Glucagon Secretion by Hyperglycemia-Induced Sodium-Dependent Reduction of ATP Production
Source: Cell Metab. 2019 Feb 5;29(2):430–442.e4. doi: 10.1016/j.cmet.2018.10.003 (PMC6370947; doi:10.1016/j.cmet.2018.10.003)
Supplement: Document S1. Figures S1–S7 and Table S1 [file mmc1.pdf]

Cell Metabolism, Volume *29*

## Supplemental Information

# Dysregulation of Glucagon Secretion by Hyperglycemia-Induced Sodium-Dependent Reduction of ATP Production

**Jakob G. Knudsen, Alexander Hamilton, Reshma Ramracheya, Andrei I. Tarasov, Melissa Brereton, Elizabeth Haythorne, Margarita V. Chibalina, Peter Spégel, Hindrik Mulder, Quan Zhang, Frances M. Ashcroft, Julie Adam, and Patrik Rorsman**

# Figure S1

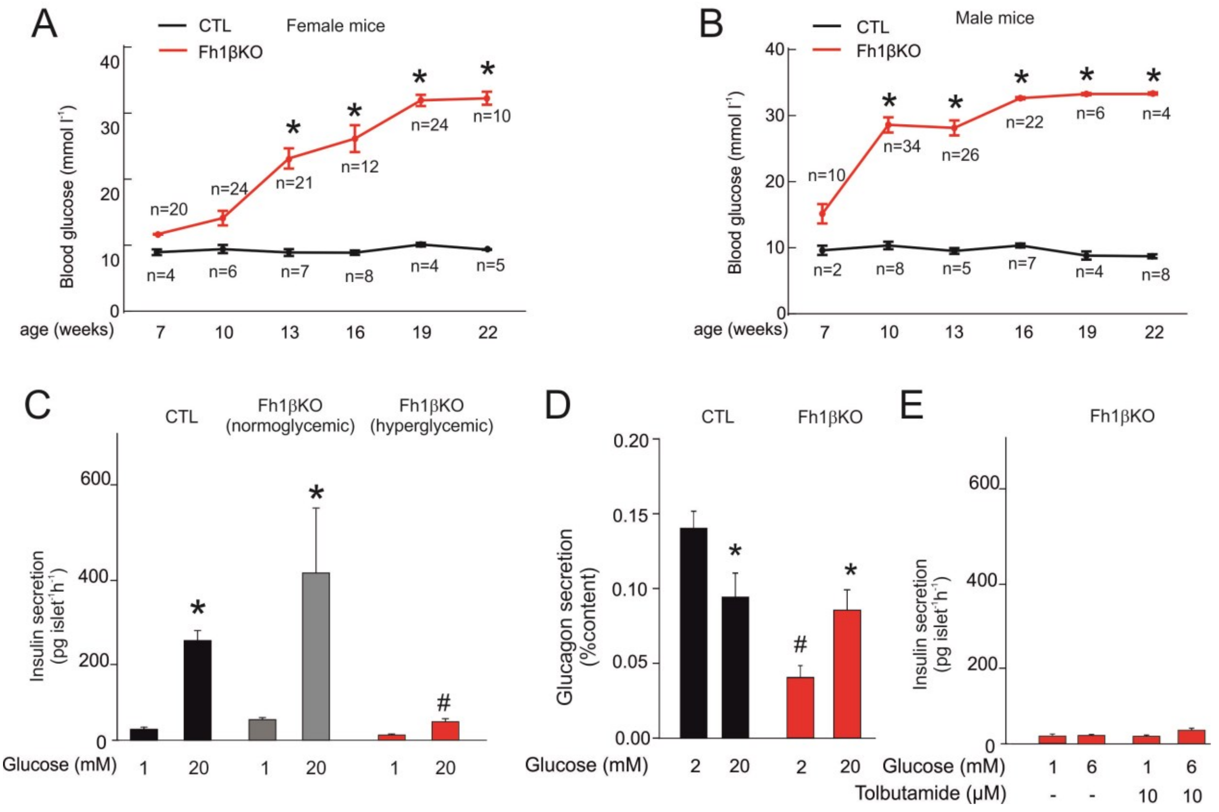


**Figure S1. Progression of diabetes in Fh1βKO mice, Related to figure 1.**

(A-B) Blood glucose in female (A) and male (B) Fh1βKO mice measured in 3-week intervals. *$P$<0.05 *vs* control. n= number of mice per time point.

(C) Insulin secretion in isolated islets from control (CTL; black), normoglycemic (plasma glucose: <12 mM; grey) and diabetic (plasma glucose: >20 mM; red) Fh1βKO mice at 1 and 20 mM glucose. *$P$<0.05 *vs* 1 mM glucose; #$P$<0.05 *vs* 20 mM glucose in normoglycemic Fh1βKO islets (n=8-9 experiments using islets from 12 mice).

(D) Glucagon secretion in islets from hyperglycemic Fh1βKO mice (red) and age-matched control mice (CTL; black) at 2 and 20 mM glucose. Data are based on 6 experiments using islets from 3 mice for each genotype. *$P$<0.05 *vs* 2 mM glucose; #$P$<0.05 *vs* 2 mM glucose CTL.

(E) Insulin secretion in islets from hyperglycemic Fh1βKO mice (red) in response to 1 or 6 mM glucose with or without 10 μM tolbutamide (n=6 experiments using islets from 6 mice).

Data are mean values ± SEM.

# Figure S2

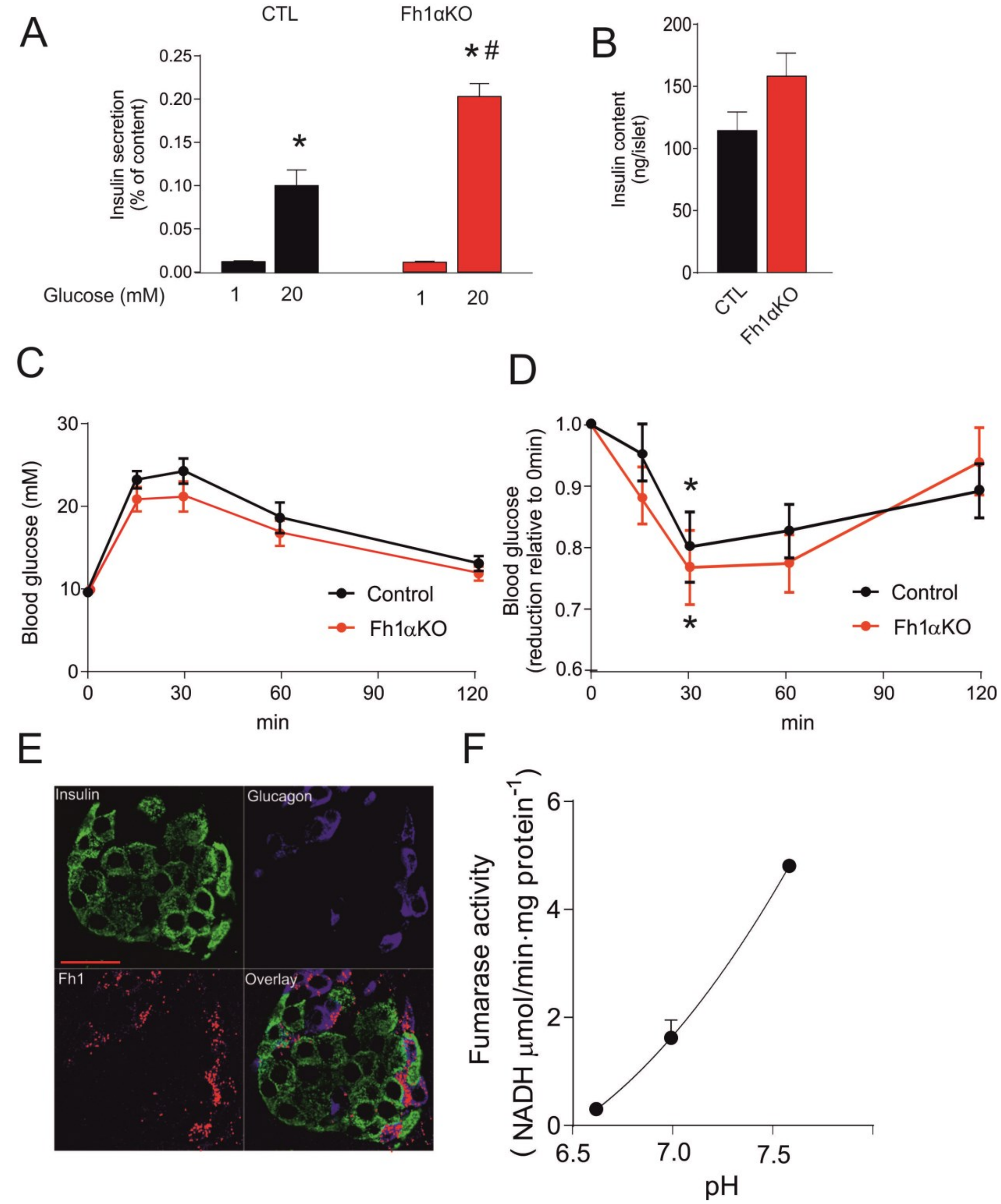


**Figure S2. Secretion and *in vivo* phenotype of Fh1αKO mice, Related to figure 2 and 4**

(A) Insulin secretion in isolated islets from control (CTL) and Fh1αKO mice at 1 and 20 mM glucose. **P*<0.05 *vs* 1 mM glucose; #*P*<0.05 *vs* 20 mM control islets (n=8-9 experiments using islets from 6 mice).

(B) Insulin content in control (CTL) and Fh1αKO mice.

(C) Glucose tolerance in control (CTL) and Fh1αKO mice; glucose tolerance was overall significantly different ($p>0.05$) in Fh1αKO mice compared with control (n=10-11 mice).

(D) Insulin tolerance in control (CTL) and Fh1αKO mice; * $P<0.05$ *vs* 0 min (n=10-11 mice).

(E) Immunofluorescence of insulin (green), glucagon (blue), Fh1 (red) and overlay of 2SC and hormones in Fh1βKO islets. Note the punctate distribution of fumarase (likely to represent mitochondria) in α-cells and lack of FH in the β-cells (in which it has been genetically ablated). Representative image of an islet from an Fh1βKO mouse (n=20 islets from 3 Fh1βKO mice). There was no overlap of glucagon and insulin (suggesting that there is no transdifferentiation; cf. (Brereton et al., 2014)). Scale bar: 50 µm.

(F) Relationship between $pH_i$ and fumarase activity (measured as NADH production from purified enzyme). The curve is a least-squares fit to the data points (n=3 experiments).

Data are mean values ± SEM.

# Figure S3

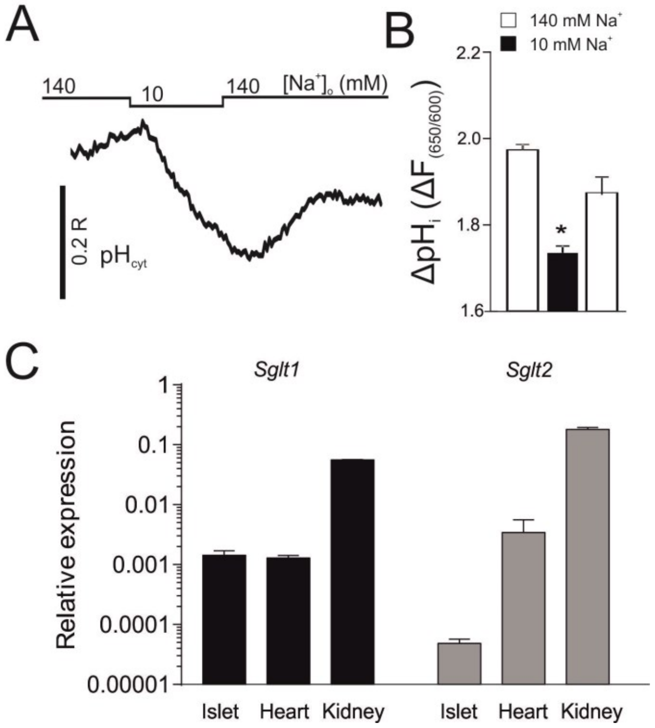


**Figure S3. SGLT-dependent acidification of α-cells, Related to figure 4.**

(A) Intracellular pH ($pH_i$) measured in wildtype mouse α-cells at normal (140 mM) or reduced (10 mM) extracellular $Na^+$ ($[Na^+]_o$) in the presence of 10 mM glucose. $[Na^+]_o$ was lowered as indicated by horizontal bar. $pH_i$ was measured using the fluorescent probe SNARF-5F and is expressed as SNARF fluorescence ratio (R), ($F_{650}/F_{600}$). Experiment performed in the presence of 10 mM glucose and 0.2 mM diazoxide (to clamp the membrane potential at ~-70 mV).

(B) Effect of extracellular sodium ($[Na^+]_o$) on $pH_i$. Mean fluorescence ratios of experiments of the type shown in (A). *$P$<0.05 *vs* initial exposure to 140 mM $[Na^+]_o$ (n=59 cells).

(C) qPCR of mRNA expression of the genes encoding the $Na^+$-dependent glucose co-transporters SGLT1 and 2 (*Slc5a1* and *Slc5a2*) in wildtype C57BL/6J islets (n=5), heart (n=5) kidney (n=3). Note use of logarithmic ordinate scale.

Data are mean values ± SEM.

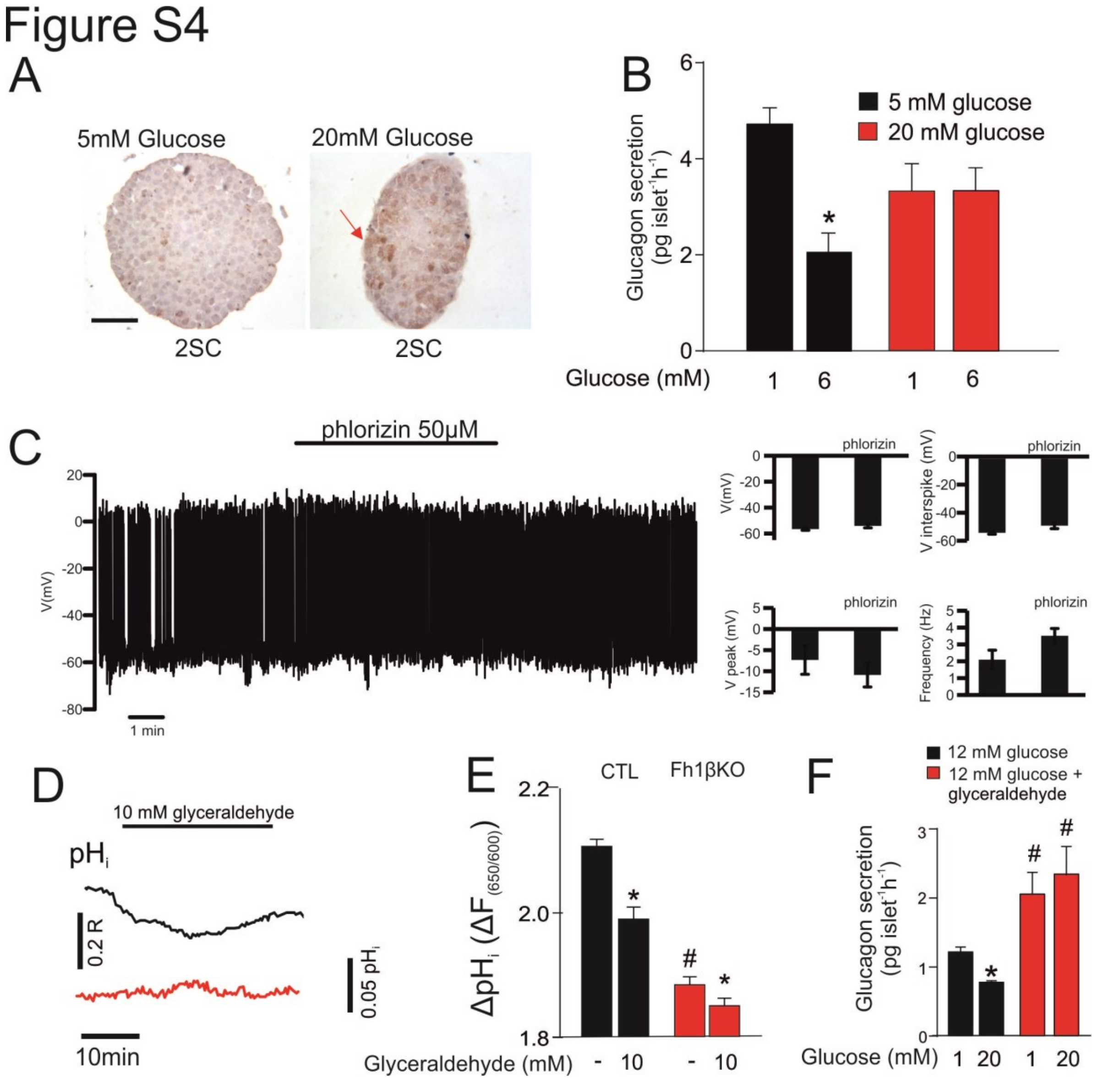


**Figure S4. Effects of chronic exposure to glucose and D-glyceraldehyde on glucagon secretion , Related to Figure 5.**

(A) IHC for 2SC in wild type islets cultured at 5 mM and 20 mM glucose for 24 h. Note 2SC labelling of peripheral islet cells (likely to be α-cells; red arrow). Representative of at least 50 islets from 3 mice.

(B) Glucagon secretion from human islets after 24 h of culture at 5 and 20 mM glucose and then testing glucagon secretion at 1 and 6 mM glucose. Data are based on 9-10 experiments using islets from 3 different donors. *$P$<0.05 *vs* 1 mM glucose.

(C) Electrical activity in α-cell exposed to 20 mM glucose with or without 50 μM phlorizin as indicated. Representative of 4 experiments from 2 mice and histograms summarizing the most repolarized interspike membrane potential (left), the peak potential (middle) and the frequency of action potentials at 20 mM glucose with/without phlorizin (4 cells: >100 action potential for each conditions and for every cell.

(D) Effect of glyceraldehyde on α-cell $pH_i$ in control (CTL) and hyperglycemic Fhβ1KO mice. $pH_i$ was measured using the fluorescent probe SNARF-5F and is expressed as SNARF fluorescence ratio (R), ($F_{650}/F_{600}$). The traces have been offset to reflect the true difference in fluorescence ratios between control and Fh1βKO α-cells.

(E) Histogram summarizing the effect of glyceraldehyde on α-cell $pH_i$ in islets from control (CTL) and hyperglycemic Fh1βKO mice.

(F) Glucagon secretion from wild type mouse islets at 1 and 20 mM glucose as indicated after 24 h of culture at 12 mM glucose supplemented with 10 mM D-glyceraldehyde. *P<0.05 *vs* 1 mM glucose. #*P*<0.05 *vs* 1 mM glucose *vs* 1 mM glucose in islets cultured at 12 mM glucose alone (n=4-6 experiments using islets from 6 mice).

Data are mean values ± SEM.

Figure S5

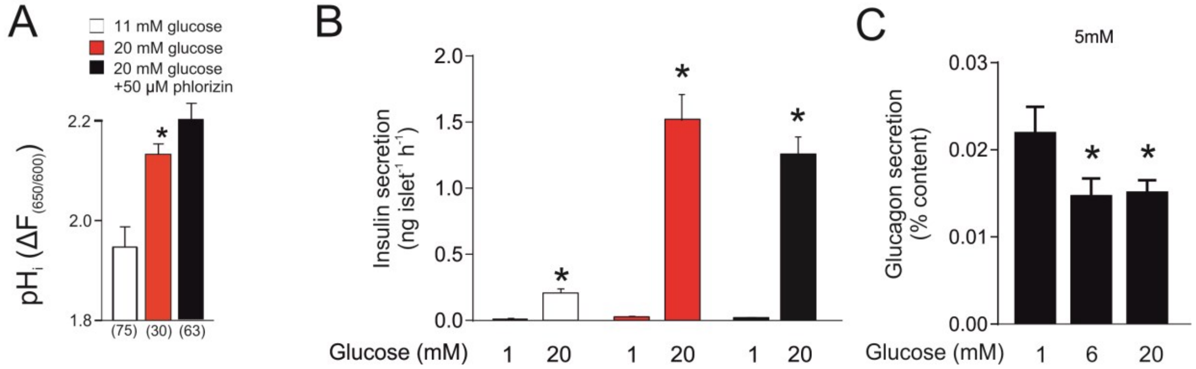


**Figure S5. Impact of chronic glucose exposure and phlorizin on β-cell $pH_i$ and glucose-induced insulin secretion, Related to figure 5.**

(A) Basal ($pH_i$) measured in β-cell in islets cultured at 11 mM or 20 mM glucose or 20 mM glucose + 50 µM phlorizin using the pH indicator SNARF. *$P$<0.05 *vs* 1 mM glucose.

(B) As in (A) but insulin secretion was measured at 1 mM or 20 mM glucose. *$P$<0.05 *vs* 1 mM glucose.

(C) Glucagon secretion during 1 h static incubation in wildtype islets incubated at 5 for 48h, at 1, 6 and 20 mM glucose as indicated. (n=6 experiments from 6 mice). *$P$<0.05 *vs* 1 mM glucose; *$P$<0.05 *vs* 1 mM glucose

Data are mean values ± SEM.

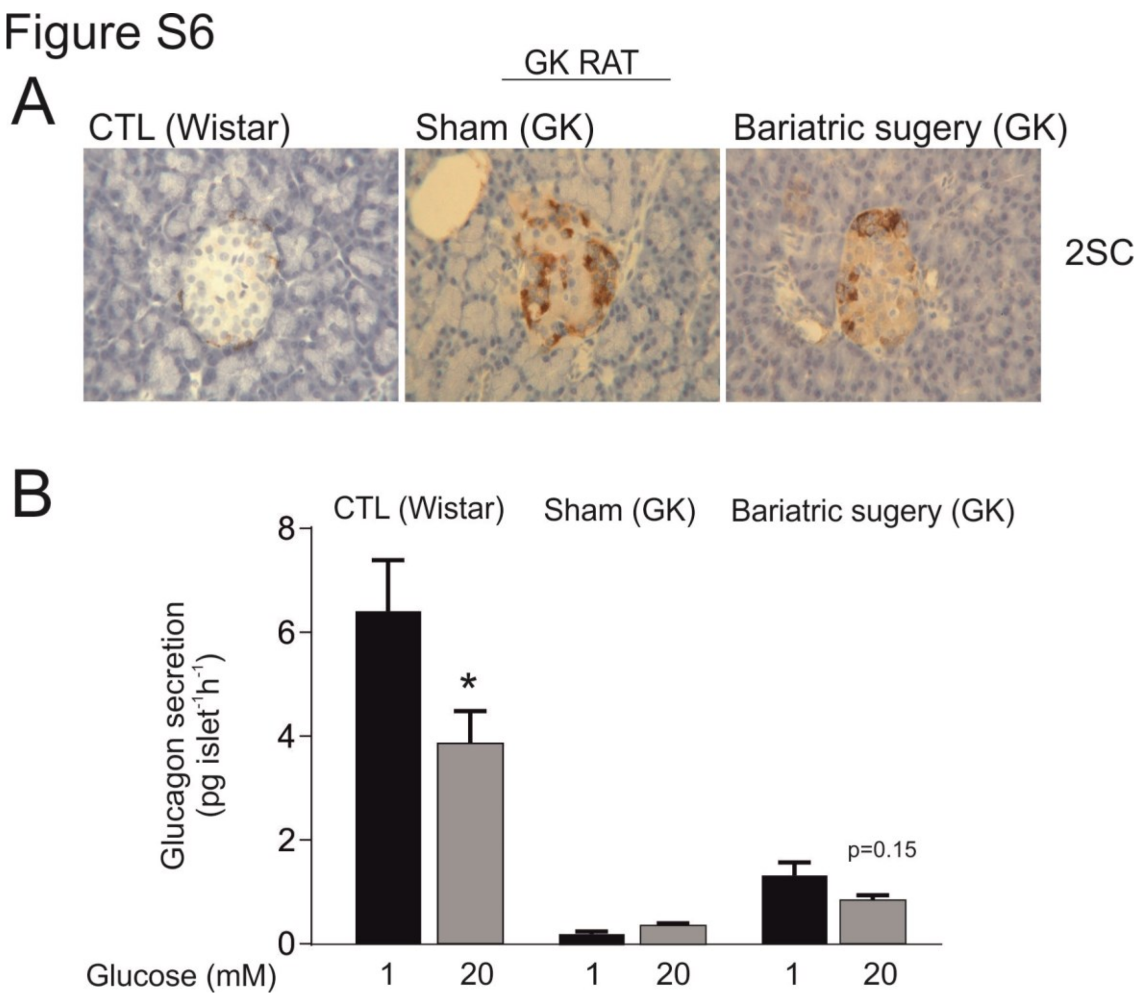


**Figure S6. Persistent protein succination and impaired glucagon secretion after restoration of normoglycemia by RYGB surgery, Related to figure 6**

(A) Pancreatic sections from Wistar (left), diabetic GK (middle), and GK RYGB (i.e. rats that had been diabetic, but in which diabetes has been resolved following RYGB surgery) immunostained for 2SC. Scale bar: 50μm.

(B) Glucagon secretion during 1 h static incubation in non-diabetic Wistar rats, diabetic GK rats and GK RYGB rats at 1 and 20 mM glucose as indicated. (n=2-5 experiments based on islets collected from >6 rats for each condition). *$P$<0.05 *vs* 1 mM glucose; p=0.15 vs 1mM glucose within GK RYGB.

Data are mean values ± SEM.

## Figure S7

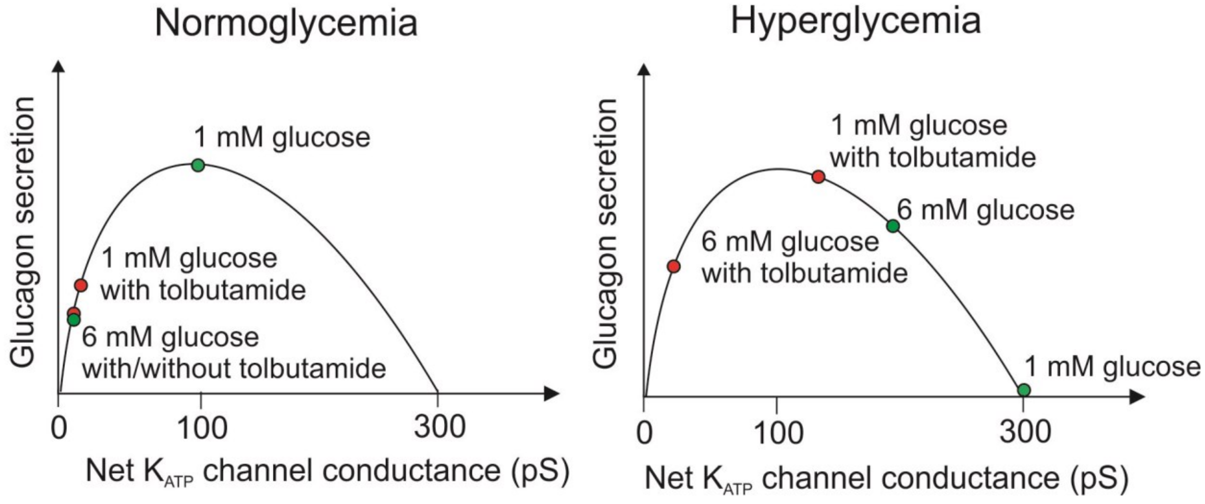


**Figure S7 Bell-shaped relationship between $K_{ATP}$ channel activity and glucagon secretion, Related to figure 3.**

A bell-shaped relationship between $K_{ATP}$ channel activity and glucagon secretion can explain the different effects of elevated glucose and tolbutamide in normoglycemic wildtype and hyperglycemic Fh1βKO and βV59M islets. $K_{ATP}$ channel activity is expressed as net conductance (in pS). Values are obtained from (Zhang et al., 2013). $K_{ATP}$ channel activity in the absence and presence of tolbutamide is represented by the green and red symbols, respectively. Under normoglycemic conditions, $K_{ATP}$ channel activity at low glucose (e.g. 1 mM) is close to that associated with maximum glucagon secretion (100 pS). Increasing glucose to 6 mM increases the intracellular ATP/ADP-ratio leading to complete inhibition of $K_{ATP}$ channel activity and glucagon secretion is inhibited because of membrane depolarization and voltage-dependent inactivation of the tetrodotoxin (TTX)-sensitive $Na^{+}$ channels. The $K_{ATP}$ channel blocker tolbutamide will also reduce $K_{ATP}$ channel activity and inhibit glucagon secretion but when used at a concentration of 10 µM it will only block 50% of channel activity (from 100 to 50 pS) resulting in partial inhibition of glucagon secretion and glucagon secretion is reduced to levels close to that seen at 6 mM glucose alone. Accordingly, increasing glucose from 1 to 6 mM in the presence of 10µM tolbutamide does not produce much further inhibition. In contrast in islets from hyperglycemic

animals, $K_{ATP}$ channel activity is slightly increased (to 300 pS; estimated from the suppression of glucagon secretion), resulting in membrane repolarization, inhibition of action potential firing and suppression of glucagon secretion (Figure 3E, right). Although an increase in glucose under these conditions will still reduce $K_{ATP}$ channel activity (by 100 pS), the suppression is not sufficient to produce a net inhibition of glucagon secretion and it may even result in a stimulation of glucagon secretion. However, when $K_{ATP}$ channel activity is first reduced by 50% by tolbutamide (i.e. to 150 pS), $K_{ATP}$ channel activity is brought into the range associated with a high rate of glucagon secretion and a further glucose-induced decrease in $K_{ATP}$ will result in inhibition of glucagon secretion.

| Subject | Age | Sex | Years from diagnosis | BMI |
|---|---|---|---|---|
| Control | | | | |
| 1 | 58 | Male | NA | - |
| 2 | 27 | Female | NA | - |
| 3 | 78 | Male | NA | - |
| 4 | 72 | Female | NA | - |
| 5 | 80 | Female | NA | - |
| 6 | 65 | Male | NA | - |
| Diabetic | | | | |
| 1 | 76 | Female | 28 | 24.6 |
| 2 | - | Male | 9 | - |
| 3 | 76 | Male | 17 | 26.1 |
| 4 | 86 | Male | 10 | 23.9 |
| 5 | 71 | Male | 8 | - |
| 6 | 66 | Male | 0.5 | - |
| 7 | 49 | Male | 6 | - |

**Supplemental Table S1. Details of non-diabetic (control) and diabetic domors used for 2SC Immunohistochemistry, Related to figure 6**

Abbreviations: NA, not applicable. BMI, body-mass index. -, information not available.
